# Genome-wide systematic characterization of the bZIP transcriptional factor family in tomato (*Solanum lycopersicum* L.)

**DOI:** 10.1186/s12864-015-1990-6

**Published:** 2015-10-12

**Authors:** Dayong Li, Fuyou Fu, Huijuan Zhang, Fengming Song

**Affiliations:** State Key Laboratory for Rice Biology, Institute of Biotechnology, Zhejiang University, Hangzhou, 310058 China; Department of Botany and Plant Pathology, Purdue University, 915 W. State Street, West Lafayette, IN 47907 USA

**Keywords:** Tomato (*Solanum lycopersicum*), bZIP transcription factor family, Phylogenetic analysis, Gene expression profile analysis

## Abstract

**Background:**

Transcription factors of the basic leucine zipper (bZIP) family represent exclusively in eukaryotes and have been shown to regulate diverse biological processes in plant growth and development as well as in abiotic and biotic stress responses. However, little is known about the bZIP family in tomato (*Solanum lycopersicum* L.).

**Methods:**

The *SlbZIP* genes were identified using local BLAST and hidden Markov model profile searches. The phylogenetic trees, conserved motifs and gene structures were generated by MEGA6.06, MEME tool and gene Structure Display Server, respectively. The syntenic block diagrams were generated by the Circos software. The transcriptional gene expression profiles were obtained using Genevestigator tool and quantitative RT-PCR.

**Results:**

In the present study, we carried out a genome-wide identification and systematic analyses of 69 *SlbZIP* genes that distributes unevenly on the tomato chromosomes. This family can be divided into 9 groups according to the phylogenetic relationship among the SlbZIP proteins. Six kinds of intron patterns (*a*–*f*) within the basic and hinge regions are defined. The additional conserved motifs and their presence of the group specificity were also identified. Further, we predicted the DNA-binding patterns and the dimerization property on the basis of the characteristic features in the basic and hinge regions and the leucine zipper, respectively, which supports our classification greatly and helps to classify 24 distinct subfamilies. Within the SlbZIP family, a total of 40 *SlbZIP* genes are located in the segmental duplicate regions in the tomato genome, suggesting that the segment chromosomal duplications contribute greatly to the expansion of the tomato SlbZIP family. Expression profiling analyses of 59 *SlbZIP* genes using quantitative RT-PCR and publicly available microarray data indicate that the tomato *SlbZIP* genes have distinct and diverse expression patterns in different tissues and developmental stages and many of the tomato *bZIP* genes might be involved in responses to various abiotic and biotic stresses as well as in response to light.

**Conclusions:**

This genome-wide systematic characterization identified a total of 69 members in the SlbZIP family and the analyses of the protein features and gene expression patterns provide useful clues for further functional characterization of the bZIP transcription factors in tomato.

**Electronic supplementary material:**

The online version of this article (doi:10.1186/s12864-015-1990-6) contains supplementary material, which is available to authorized users.

## Background

Transcription factors (TFs) are key regulators of numerous signaling networks in response to growth and development as well as to environmental stresses through binding to promoters of specific sets of target genes to activate or repress their expression. Among the TF families, the basic leucine (Leu) zipper (bZIP) of TF family is one of the largest and most diverse families [1]. The bZIP TFs are named according to their common feature, bZIP domain, which consists of ∼ 60–80 amino acids in length, surrounded by two functionally distinct regions, a basic region and a Leu zipper [1]. In bZIP proteins, the basic region of around 18 amino acid residues with an invariant motif N-x_7_-R/K-x_9_ is responsible for nuclear localization and DNA binding, whereas the following Leu zipper motif made up of several heptad repeats of Leu or other bulky hydrophobic amino acids (e.g., Ile, Val, Phe or Met) is less conserved and mediates the homo- and/or heterodimerization [2]. Plant bZIP proteins harbor a relaxed binding specificity for DNA sequence motifs containing an ACGT core, and preferentially bind to the G-box (CACGTG), C-box (GACGTC) and A-box (TACGTA) [3]. At the time of DNA binding, the N-terminal half of the basic region inserts into the major groove of double-stranded DNA and the C-terminal half of the Leu zipper mediates dimerization to form a superimposed coiled-coil structure [2, 4].

With the completion of sequencing of many eukaryotic genomes, members of the bZIP TF family have been identified or predicted at genome-wide level. The numbers of the bZIP TF family vary among the organisms examined so far. For example, it was reported 17 members in *Saccharomyces cerevisiae* [5], 27 in *Drosophila* [6] and 56 in humans [7]. Similarly, relative large numbers of the bZIP TF family in various plants were identified, e.g. 75 in Arabidopsis [8], 49 in castor bean [9], 64 in cucumber [10], 89 in rice [11], 125 in maize [12], 92 in sorghum [13], 89 in barley [14], 131 in soybean [15], 55 in grapevine [16] and 96 in *Brachypodium distachyon* [17]. However, only a small portion of the bZIP TFs has been studied at biochemical, molecular and functional levels for the biological functions in plants. Extensive studies through knockout/knockdown or overexpression approaches in model plant species demonstrated that members of the bZIP TF family participate in the differentiation of many organs and tissues, embryogenesis, seed maturation, floral transition and initiation and vascular development in plants [8, 18, 19]. In addition, the bZIP TFs have also been shown to act as key components in the signaling pathways that mediate responses to abiotic and biotic stresses such as osmotic, hypoxia, drought, high salinity and cold stresses, and pathogen infection [5, 18–21].

In tomato, only a few of the bZIP TFs have been identified and functionally characterized. The best-studied tomato bZIP TF, SlAREB1 (abscisic acid-responsive element binding protein 1), was shown to play important roles in response to environmental stress and metabolic programming during fruit ripening and also participate as a link of ABA signaling to biotic stress responses [22–26]. VSF-1, a development-related bZIP member, was found to bind the promoter of *GRP1.8*, which encodes a glycine-rich structural protein in cell wall, and specifically regulate its expression in vascular tissue [27, 28]. Expression of some bZIP genes such as *SlAREB2*, *ABZ1* and *LebZIP1* were shown to be induced by drought, salt and anaerobic stresses and wounding or by organ-specific signals [24, 29, 30]. Collectively, information on the tomato bZIP TF family and their biological functions is quite limited and therefore genome-wide systematic characterization of the bZIP family is a priority for detailed functional studies of this important family in tomato.

The tomato genome has recently been completely sequenced and the genome database is freely available to the scientific community. This provides an excellent platform, offering an opportunity to characterize gene families at the genome-wide level. In the present study, we performed a genome-wide systematic characterization of the tomato bZIP (SlbZIP) family. As a consequence, a total of 69 members were identified in the SlbZIP family. Details on the protein domain organization, gene structure, chromosome distribution, phylogenetic tree analyses and evolution were also presented. Furthermore, the spatial and temporal expression patterns of selected members of the SlbZIP family during various developmental stages and in response to nutrition status, abiotic and biotic stress were also analyzed using publicly available microarray expression data. This study provides important starting points to further study the biological functions of the SlbZIP family in tomato.

## Results and discussion

### Characterization and nomenclature of the SlbZIP family

Based on an extensive survey against tomato genome database using the conserved bZIP domain sequence as a BLASTP query, a total of 104 putative SlbZIP candidates were initially obtained with the *E*-value threshold of 1.0. After further database searching and alignment with known bZIP proteins from other plants, a total of 69 non-redundant SlbZIP TFs were identified. For further convenience, we assigned unique names to these SlbZIPs as SlbZIP1-69 according to the previously proposed nomenclature system [8, 11, 31] (Additional file 1: Table S1). Compared with other plants, the tomato SlbZIP family is comparable to Arabidopsis (75 members) [8], relatively smaller than rice (89 members) [11], maize (125 members) [12], sorghum (92 members) [13], barley (89 members) [14], soybean (131 members) [15] and *B. distachyon* (96 members) [17] but larger than castor bean (49 members) [9], cucumber (64 members) [10] and grapevine (55 members) [16]. It seems likely that the monocot plants harbor a relatively larger bZIP family than the dicot plants, probably due to that the higher number of bZIP members evolved in monocots than in dicots after the divergence of monocots from dicots [9, 13]. Furthermore, the sequenced tomato ‘Heinz 1706’ genome, which is approximately 900 Mb in size, is 7.2 times larger than the Arabidopsis genome (~125 Mb in size). However, the number of the *SlbZIP* genes was similar to Arabidopsis. According to the predicted total genes, the ratio to the SlbZIP family in the tomato genome was estimated to be about 0.20 %, which is less than Arabidopsis (0.27 %). Searches against the SOL Unigene and NCBI cDNA databases identified putative full-length cDNAs corresponding to 59 out of 69 *SlbZIP* genes (Additional file 1: Table S1), indicating that most of the annotated *SlbZIP* genes are expressed in tomato.

### Phylogenetic analysis and classification of the SlbZIP family

**Fig. 1:**
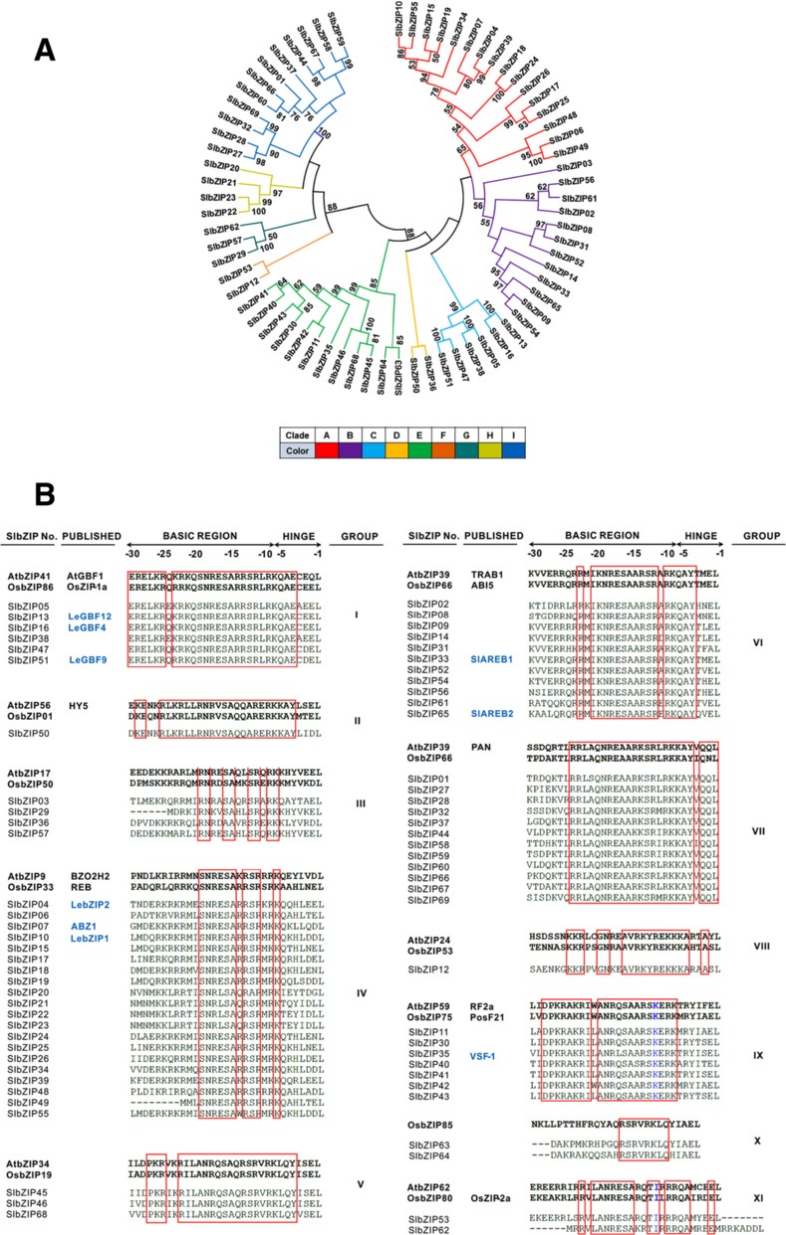
Phylogenetic relationship and alignment of basic and hinge regions of the SlbZIP proteins. **a** The phylogenetic tree is based on the sequence alignments of the SlbZIP proteins. Dendrogram constructed using Neighbor-Joining method by MEGA6.06. Bootstrap values from 1000 replicates are indicated at each node. Only bootstrap values larger than 50 % support are indicated. SlbZIP proteins are grouped into 9 distinct clades (A-I). **b** Alignment of basic and hinge regions of SlbZIP proteins. The first Leu of the Leu zipper was regarded as +1, and asparagine and arginine were numbered −18 and −10, respectively. Two already characterized plant bZIP proteins are shown on the top of each group for comparison. Amino acid residues K and I belonging to group IX and XI, respectively, are colored in blue as they differ from the usual R at −10 positions. SlbZIP gene names and protein names of already characterized SlbZIP proteins are also given on the left hand side

To analyze the evolution of the *SlbZIP* genes, an unrooted phylogenetic tree was generated using the sequence alignments of the SlbZIP proteins. As shown in Fig. 1a, the 69 *SlbZIPs* could be clustered into nine clades with well-supported bootstrap values and these 9 clades, namely A to I comprise of 16, 12, 6, 2, 12, 2, 3, 4 and 12 proteins, respectively. Compared with the number of clades in the other plant species, the tomato SlbZIP family has same number of clades with castor bean [9], less clades than rice and maize, which both have 10 clades [11, 12], but more clades than cucumber and sorghum, which have 6 and 7 clades, respectively [10, 13]. According to the DNA-binding specificity, the 69 SlbZIPs could also be categorized into 11 groups (I–XI) (Fig. 1b). It was observed that a majority of the members, predicted to have similar DNA-binding properties, clustered together into same clades (Fig. 1a, b). By contrast, certain members in groups III, IV, V and XI were clustered apart into different clades. For example, all the six members in group I and 11 members in group VI were clustered into clade C and B, respectively; whereas 12 members in group VI and 7 members in group IX were clustered into clade B and E, respectively. In addition, 19 members in group IV were separately distributed in clades A, B and H.

To analyze the relationships of SlbZIPs at the amino acid level, three different phylogenetic trees were generated based on the bZIP domain, basic and hinge regions and Leu zipper region, respectively (Additional file 2: Figure S1). These three phylogenetic trees show high similarity of trends in grouping the SlbZIPs into different clades. The only difference is that SlbZIP36 and SlbZIP50 are clustered into different clades in the Leu zipper tree while they are in the same clade in the bZIP domain and the basic and hinge region trees (Additional file 2: Figure S1).

To elucidate the phylogenetic relationships of bZIPs among tomato, Arabidopsis and rice, another unrooted phylogenetic tree was constructed (Additional file 3: Figure S2). Notably, most of the clades contain tomato, Arabidopsis and rice bZIP proteins, indicating that at least part of the bZIPs appeared before divergence between monocots and dicots. The interspecies clustering shown in the tree also suggests the existence of homologous *bZIP* genes among tomato, Arabidopsis and rice. For example, the predicated G-box binding SlbZIP proteins, which belong to Group I, are clustered together with other known G-box binding bZIP proteins form Arabidopsis and rice, e.g. AtGBF1/2/3 [32], OSBZ8 [33] and OsZIP1-a [34] (Additional file 3: Figure S2). Another example is that the SlbZIP proteins with predicated ABRE-binding feature are also clustered together with rice TRAB1 and Arabidopsis ABF1/3/4, ABI5 and DPBF2/4 [35, 36]. These phylogenetic analyses indicate that the structure and function of bZIPs are probably conserved across plant species during evolution.

### Features and structure of the SlbZIP proteins

**Fig. 2:**
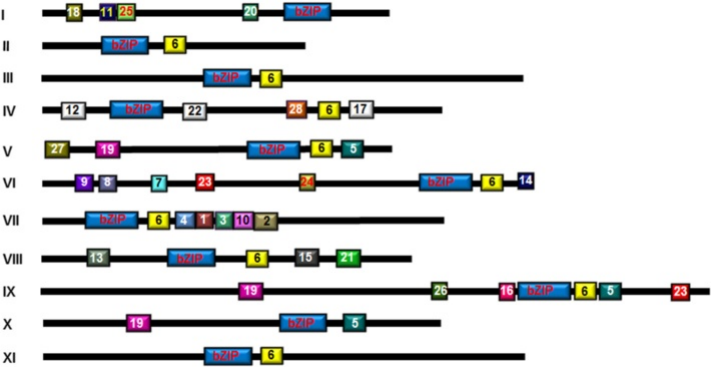
Distribution of additional conserved motifs identified by MEME. The bZIP domains are shown in blue. Different motifs are highlighted in different color boxes with numbers 1 to 28. The details of predicted conserved motifs are given in Additional file 4: Table S2

Generally, the SlbZIP proteins range from 124 to 660 amino acids (aa) in sizes (318 aa in average) with a range of molecular weights from 13.40 kDa to 70.98 kDa (Additional file 1: Table S1). The sizes of the SlbZIP proteins are similar to those in *Arabidopsis* (321 aa in average) [8] and rice (311 aa in average) [11]. All these SlbZIP proteins contain one typical bZIP domain, although the locations of the bZIP domain within the SlbZIP proteins vary greatly (Fig. 2).

In addition to the bZIP domain, further searches for the presence of other conserved motifs identified a total of 28 additional conserved motifs in 69 SlbZIP proteins (Fig. 2). It is observed that most of the SlbZIP proteins clustered in the same clade share one or more conserved motif outside the bZIP domain (Fig. 2). The details of these conserved motifs are listed in Additional file 4: Table S2. It was found that some motifs are shared by several groups, such as motif 6 in 9 groups, motif 19 presents in 3 groups and motif 5 and motif 23 present in 2 groups. However, most of the conserved motifs appear in specific groups, implying that the group-specific motifs may determine the specific function for the members in these groups [11, 12]. For example, motif 20 in group I was found to be a part of the transactivation domain, conserved among plant HBP-1a/GBF-type bZIP factors [37, 38]. Motifs 7, 8, and 9 contain conserved TLED/E, TVDE and T(L/F)DE and parts of them represent potential casein kinase II (CKII) phosphorylation sites (S/TxxD/E), which have been reported to be present in some Arabidopsis ABF (ABRE-binding factor) and AREB (ABA-responsive element-binding protein) [35, 36, 39, 40]. Motif 24 also contains a phosphorylation site (R/KxxS/T), presented as [KR][SY][CGS][ST], for Ca^2+^-dependent protein kinase and has been identified in members of group VI bZIPs in Arabidopsis, including AREBs/ABFs [8, 41]. SlbZIP34 (SlAREB1) and SlbZIP67 (SlAREB2), belonging to group VI, have been experimentally verified to function as ABA-dependent TFs that positively modulate abiotic stress tolerance and regulate the metabolic programming during fruit ripening [22, 24]. Motif 11, characterized by a part of the proline-rich domain, is present exclusively in group I, which has been shown to have transcriptional activation potential [42].

Among the 11 groups, group VI has the largest number of unique motifs and most of the members in this group share motifs 6, 7, 8, 9, 14, 23 and 24. It is thus speculated that the multifunction of the group VI members such as *SlAREB1* and *SlAREB2* in regulating abiotic stress responses and development of tomato might be due to these unique motifs [43, 44]. Further analysis and comparison also revealed some common motifs among tomato, rice and maize bZIPs in different groups. For example, motifs 1, 2 and 3 in tomato group VII are the same as motifs 18, 20 and 19 in rice and motifs 1, 2 and 5 in maize, respectively; whereas motif 16 in tomato group IX is the same as motif 25 in rice and motif 9 in maize [11, 12].

### Prediction of DNA-binding site specificity of SlbZIPs

Previous reports demonstrated that both of the core basic region and the hinge region determine the binding specificity of bZIP TFs [45, 46]. To predict the DNA-binding site specificity of the SlbZIP proteins, amino acid sequences of the basic and hinge regions from 69 SlbZIPs were aligned (Fig. 1b). Based on the alignment and the type of amino acid residues present in the basic and hinge regions, SlbZIP could be categorized into 11 groups, named I-XI (Fig. 1b) [46]. The characteristic features of the SlbZIP proteins classified into different groups are described in Additional file 5: Table S3. As shown in Fig. 1b, each group has highly conserved amino acid residues in the basic and hinge regions. It was previously reported that the amino acid replacement at certain sites in basic and hinge regions can affect the DNA-binding specificities [47]. In the tomato SlbZIP family, the amino acid replacements in basic and hinge regions were only detected in group IX and XI. SlbZIP53 and SlbZIP628 in group XI have a hydrophobic Ile at position −10 instead of Arg/Lys, implying that they may not be able to bind DNA or possess a unique DNA-binding specificity. All the members of group IX have a conserved Lys substitution at position −18 instead of Arg, implying a different requirement for dimerization.

### The Leu zippers and dimerization property in SlbZIPs

**Fig. 3:**
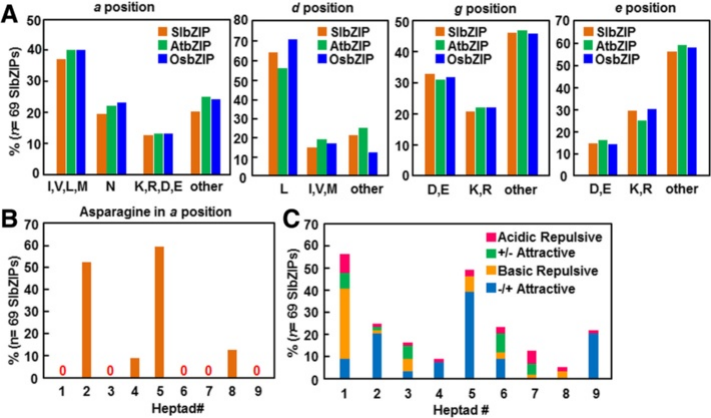
Amino acid analysis at the *g*, *e*, *a*, and *d* positions of the Leu zipper and frequency of attractive or repulsive *g*-*e*’ pairs. **a**, Histogram of frequency of amino acids in the *g*, *e*, *a*, and *d* positions of the Leu zipper for the SlbZIP, AtbZIP, and OsbZIP proteins. **b**, Histogram of the frequency of Asn residue in the *a* position of the Leu zippers for all SlbZIP proteins. **c** Histogram of the frequency of attractive or repulsive *g*-*e*’ pairs per heptad for all SlbZIP proteins. Complete *g*↔*e*’ pairs were defined if both amino acids at the *g* and the following *e* positions are charged while incomplete *g*↔*e*’ pairs were defined if only one of the amino acids at the *g* or *e* position is charged. According to the electrostatic charges at the *g* and *e* positions, the complete *g*↔*e*’ pairs were classified into four groups, namely acidic repulsive (acidic amino acids at the *g* and *e* positions), basic repulsive (basic amino acids at the *g* and *e* positions), +/−attractive (acidic amino acid at *g* position and basic amino acid at *e* position) and −/+attractive (basic amino acid at *g* position and acidic amino acid at *e* position)

The amino acid sequence of the Leu zipper region of bZIP domain is known to determine the homo- and/or heterodimerization of the bZIP proteins [48]. The amino acids at the *a*, *d*, *e* and *g* positions play important roles in regulating the oligomerization, dimerization stability and specificity of the Leu zippers because of their specific locations near the Leu zipper interface [49, 50]. Based on the presence of attractive or repulsive interhelical *g*↔*e* electrostatic interactions and the presence of polar or charged amino acids at the *a* and *d* positions of the hydrophobic interface, the bZIP dimerization specificity was predicted in bZIP proteins from Arabidopsis, rice, maize and cucumber [6, 7, 49]. To predict the dimerization specificity of the SlbZIP proteins, we analyzed the type of amino acids at the *a*, *d*, *e*, and *g* positions. As shown in Fig. 3a, hydrophobic amino acids are predominant at the *a* and *d* positions, accounting for 84 % of the total. Approximately 19 % of amino acids at the *a* position are Asns (Fig. 3a), which is lower than the amount in Arabidopsis (22 %) and rice (23 %) [8, 11], indicating a lower number of homodimerized Leu zippers in SlbZIPs, as N-N interhelical interaction is preferred over interaction with other amino acids at the *a* position. Specifically, high frequencies of Asn at the *a* position both in the second and fifth heptads were detected, accounting for 52.2 and 59.4 %, respectively (Fig. 3b). This is similar to the observation in maize [12] but differs from those in rice and Arabidopsis, which have the highest frequency of Asn at the *a* position in the second fifth heptads [8, 11]. Additionally, the fourth and eighth heptads in SlbZIPs contain Asn with the frequencies of 8.7 and 12.3 %, respectively (Fig. 3b). The high frequency of Asn at the *a* position implies that a quite number of homodimerized Leu zippers may be formed among the SlbZIP proteins, because asparagines produce more stable N-N interactions at the *a*↔*a*’ positions than other amino acids at the *a* position [51]. On the other hand, charged amino acids at the *a* positions, which drive heterodimer formation [7], were also found in SlbZIPs. Notably, the frequency of stabilizing Leu at the *d* positions in SlbZIPs is approximately 64 %, which is higher than that in Arabidopsis (56 %) but is lower than those in rice (71 %) and maize (70 %) [8, 11, 12].

Charged amino acids occupy almost half of the *e* and *g* positions, with frequencies of 43 % and 54 %, respectively. As shown in Fig. 3c, the maximum frequency of the complete *g*↔*e*’ pairs is 26.3 %, appeared in the first heptad, among which the attractive *g*↔*e*′ pairs are in the majority (56 %); whereas the minimum frequency of the complete *g*↔*e*’ pairs is 2.3 %, appeared in the eighth heptad that has only two repulsive *g*↔*e*’ pairs. The frequencies of the complete *g*↔*e*’ pairs in the 2nd, 3rd and 4th heptads, representing approximately 11.5, 7.4 and 4.1 %, respectively, decrease significantly (Fig. 3c). But in the fifth heptads, the frequency of complete *g*↔*e*’ pairs increases, reaching to 22.6 %, in which −/+attractive *g*↔*e*’ pairs occupies 80 % (Fig. 3c). Moreover, the ninth heptad of the tomato SlbZIPs also has basic repulsive besides −/+attractive (Fig. 3c), different from the observations that only ± attractive are present in the ninth heptads in OsbZIPs and ZmbZIPs [11, 12]. Notably, a few SlbZIP proteins such as SlbZIP21-24 and SlbZIP63, have multiple repulsive *g*↔*e*’ pairs, which is similar to the observations in rice and maize bZIP proteins [11, 12] but is completely absent in Arabidopsis bZIP proteins [8]. Furthermore, there are 37.8 % of the *g*↔*e*’ interactions containing single-charged amino acids in tomato SlbZIPs, which is higher than that in maize bZIPs (32 %) [12]. In general, these Leu zippers with incomplete *g*↔*e*’ pairs contribute little to the stability of the homodimer, but they can form complete attractive *g*↔*e*’ interactions and contribute to stability through complementation in a heterodimer.

Based on the analyses of the dimerization properties described above, the 69 SlbZIP proteins were classified into 24 subfamilies (BZ1–BZ24) (Additional file 6: Figure S3 and Additional file 7: Table S4). Subfamilies BZ1, BZ3 and BZ23 tend to form homodimerization because of the appearance of attractive *g*↔*e*’ pair in the first heptad but absence of any repulsive interaction (Additional file 6: Figure S3). Subfamilies BZ18, BZ19 and most members of BZ17 have heterodimerizing specificity as they only contain repulsive interhelical interactions (Additional file 6: Figure S3). The rest of the subfamilies have both homo- and heterodimerization properties. It is thus concluded that the SlbZIP proteins have complex and varied dimerization patterns with potential to homodimerize with themselves or members from the same subfamily as well as heterodimerize with members from other subfamily. Interestingly, length of the Leu zippers in the SlbZIP family varied, ranging from three to nine heptads. Of the SlbZIP proteins, 21 % have only three short Leu zippers (BZ1 and BZ24) and more than 21 % have no α-helix breakers for 10 or more heptads (BZ10, BZ15-BZ17 and BZ22).

Interactions between several *A. thaliana* bZIP proteins were previously reported [49]. For example, deletion analysis demonstrated that the short leucine zippers with charged amino acid at the *a* positions of the first 3 heptads in AtTGA proteins, which belong to subfamily T of the bZIP family, can destabilize the leucine zipper structure [49, 52]. This is consistent with the observation that the leucine zippers of the TGA proteins, which have multifunctional roles in plant defense, xenobiotic stress and development [8, 20], are unstable (Additional file 7: Table S4).

### Structure of the *SlbZIP* genes

**Fig. 4:**
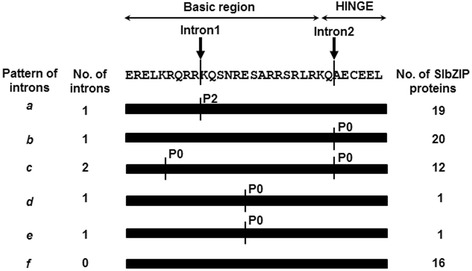
Intron patterns within the basic and hinge regions of the bZIP domains in SlbZIP proteins. SlbZIP genes are divided into six patterns (a–f) on the basis of the intron number, position and splicing phase. The arrows on the top example sequence indicate the position of two introns. The number of introns and the number of SlbZIP proteins having a particular pattern are also indicated. Phase 0 (P0) and Phase 2 (P2) indicate the splicing phases of the basic and hinge regions of the bZIP domains. P0 represent the intron splicing site between codons, P2 means the intron splicing site locating between the second nucleotide and the third nucleotide in one codon

Genomic structure of each gene may be an imprint that records key events during evolution and thus provides insights into understanding the emergence and evolution of a given gene and even a given gene family [53]. To gain insights into the structural evolution of the *SlbZIP* genes, their exon-intron organizations were analyzed based on the categorized I-XI groups (Fig. 4 and Additional file 8: Figure S4). Among the 69 *SlbZIP* genes, 12, accounting for 17.39 % of the family, are intronless and all of them belong to group IV. Similar percentages of the intronless genes in the bZIP family were observed in Arabidopsis, cucumber, rice, maize and sorghum [8, 10–13]. On the other hand, a great difference in the number of introns, ranging from 1 to 11, within the open reading frame (ORF) of the intron-containing *SlbZIP* genes was detected. Among these intron-containing genes, the intron numbers in the *SlbZIP* genes belonging to groups I and VII show the greatest degree of variation, both with 7–11, whereas the *SlbZIP* genes in the rest groups mostly have 1–3 introns (Additional file 8: Figure S4). Interestingly, all *SlbZIP* genes in groups 2, 5 and 6 and most members in groups 7 and 17 have 3 introns (Additional file 8: Figure S4).

The intron positions within the ORF are diverse and the phases of the splicing sites differ from each other; however, the positions and phases of introns in the basic and hinge regions of the bZIP domain are highly conserved [31]. Among the 57 intron-containing SlbZIP genes, accounting for 83 % of the family, 53 have introns in the bZIP domain region. Six intron patterns, namely *a* to *f*, in the bZIP domain region were identified according to the intron position, number and splicing phase within the basic and hinge regions (Fig. 4, Additional file 9: Figure S5 and Additional file 1: Table S1). This intron patterns is similar to those in maize [12] and barley [14] but different from those in rice (7 patterns) [11], grapevine (9 patterns) [16] and castor bean [9]. Among six intron patterns, *a* and *b*, which have a single intron and are typical of 19 and 20 *SlbZIPs*, respectively, are mostly represented. Pattern *a* occurs in most of the groups while pattern *c* was only found in group VII SlbZIPs (Additional file 9: Figure S5). Two introns were observed in pattern *c* and both have P0 splicing phase model. Patterns *d* and *e*, each representing one SlbZIP in group X, have one intron with same position and P2 splicing phase model. The interrupted amino acid residues are different in patterns *d* and *e*, e.g. interruption of Gln in pattern *d* while interruption of Arg in pattern *e*. Pattern *f* does not have intron in the basic and hinge regions and contains 16 *SlbZIPs* (Fig. 4 and Additional file 9: Figure S5). Among them, 12 members are intronless, while the remaining 4 have introns outside the basic and hinge regions.

### Chromosomal distribution and evolution analysis of the bZIP gene family

**Fig. 5:**
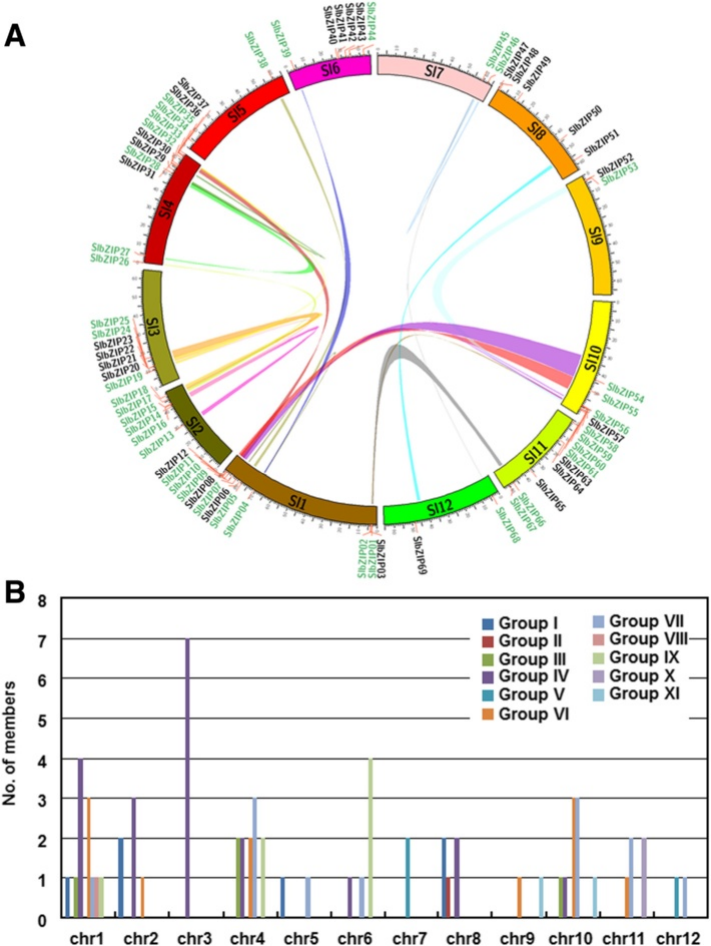
Distribution and segmental duplication of SlbZIP genes on tomato chromosomes. **a** Segmental duplication regions were determined using the SyMAP database. Genes and segmental duplication regions were mapped to the tomato chromosomes via the Circos tool. The tomato chromosomes were arranged in a circle. Ribbon links represent segmental duplication regions. The genes name located in segmental duplication regions were colored in green. **b** Histogram of all SlbZIP genes distribute on the tomato chromosomes. Different color represents *SlbZIP* genes from different group

According to the annotation and the chromosomal distribution, the 69 SlbZIP genes were mapped on all 12 tomato chromosomes with relatively high variable densities, varying from 2 to 12 among chromosomes (Fig. 5a, b). Chromosomes 1 and 4 contain the largest numbers of *SlbZIPs* with 12 and 11 members, respectively, whereas only two *SlbZIP* genes are present on chromosomes 5, 7, 9 and 12 (Fig. 5a, b). Furthermore, uneven chromosomal distribution of *SlbZIP* genes in different groups was also observed (Fig. 5a, b). For example, *SlbZIPs* in group X show a preferential distribution on chromosome 11, whereas only *SlbZIPs* in groups IV and V distribute on chromosome 3 and 7, respectively. However, *SlbZIPs* in group IV distribute more evenly than other groups across all chromosomes. This uneven chromosomal distribution of the *SlbZIP* genes in different group is similar to the maize *ZmbZIPs* and cucumber *CsbZIPs* [10, 12].

Tandem duplication and segmental duplication are important events that drive the evolution and expansion of gene family and protein functional diversification [54]. Based on the previous genomic analysis on tomato [55], the occurrence and contribution of tandem duplication and segmental duplication in the evolution of the SlbZIP family were analyzed. Generally, gene cluster is one of the results of gene tandem duplication [56]. A total of 8 *SlbZIP* gene clusters, composing of 21 *SlbZIP* genes (*SlbZIP01*-*SlbZIP02, SlbZIP10*-*SlbZIP11, SlbZIP20*-*SlbZIP24, SlbZIP30*-*SlbZIP31, SlbZIP35*-*SlbZIP34, SlbZIP53*-*SlbZIP52, SlbZIP56*-*SlbZIP58* and *SlbZIP59*-*SlbZIP61*) were identified in the tomato genome (Additional file 10: Table S5). In addition, 28 segregation duplication events were also identified (Fig. 5a and Additional file 10: Table S5).

The segmentally duplicated chromosome blocks in tomato have been identified at a genome-wide level [55]. To analyze the possible relationship between *SlbZIP* genes and potential gene duplication within the genome, we analyzed the occurrence of tandem duplication and large-scale segmental duplication during the evolution of the *SlbZIP* family. To view whether the *SlbZIP* genes are located on the syntenic duplicated segmental regions, the syntenic blocks on each chromosome and the distribution map of the *SlbZIP* genes on each chromosome were analyzed (Fig. 5). Forty *SlbZIP* genes located on the duplicated segmental regions of tomato chromosomes were identified (Additional file 11: Table S6). Interestingly, these collinear pairs of *SlbZIP* genes located on the syntenic regions belong to the same corresponding groups (Fig. 1b). Among them, 13 members of group IV constitute the most collinear gene pairs (13 of 29), accounting for 45 %, and all the group V members and more than 65 % of the group I (4 of 6) and group VII (10 of 12) members were found to be present on the duplicated segments of tomato chromosomes. Twenty seven of these *SlbZIP* genes segmentally duplicated once and the rest duplicated more than once. Surprisingly, 7 of 20 group IV members locate in syntenic regions that were segmentally duplicated at a high frequency (Additional file 11: Table S6). By contrast, no member in groups II, III, VIII and X was found to be located in the syntenic duplicated region, implying that these groups might evolve after the emergence of large-scale segmental duplication events. Thus, it is likely that the expansion of the *SlbZIP* family might be the consequence of segmental chromosomal duplication and rearrangement events rather than the independent duplication of individual sequences. Similar evolution mechanism was also found in the bZIP family in rice, *Arabidopsis* and sorghum [8, 11, 13]. Furthermore, 14 out of 27 gene pairs identified (Additional file 11: Table S6) are located on the duplicated segmental regions of tomato chromosomes, suggesting the existence of unidentified duplicated chromosomal segments on tomato chromosomes.

### Development- and tissue-specific expression of the *SlbZIP* genes

Increasing evidence has shown that *bZIP* genes are widely involved in the growth and development of higher plants [19]. However, the involvement of *bZIP* genes in the regulation of tomato growth and development is less known except that SlAREB1 was reported to regulate primary metabolic pathways in tomato fruits [22].

**Fig. 6:**
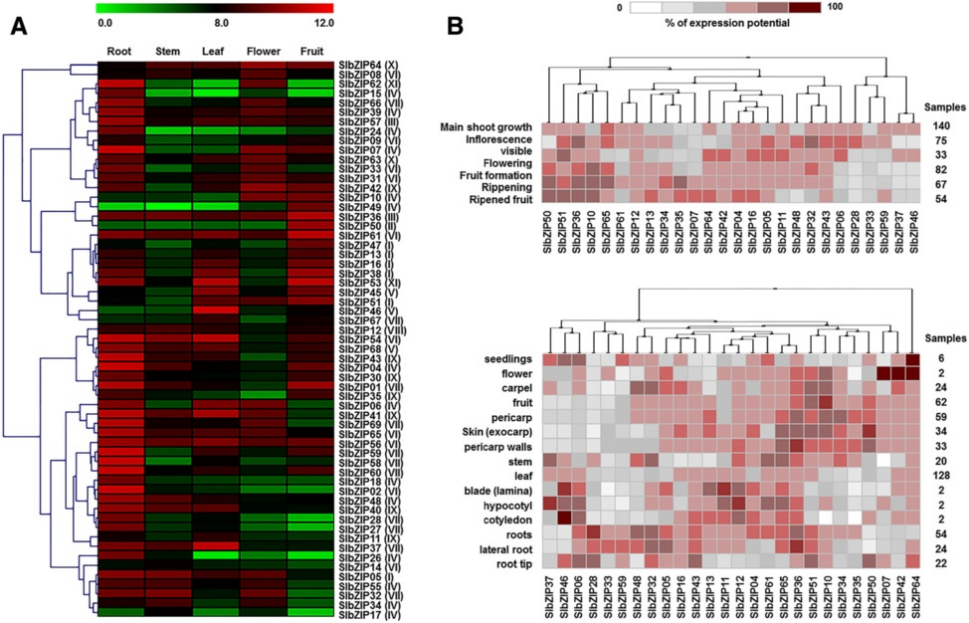
Expression patterns of *SlbZIP* genes in different developmental stages and tissues. **a** Expression profiles of *SlbZIP* genes in different organs/tissues using qRT-PCR analysis. All samples were run in triplicate and the data were normalized relative to the *SlActin* (accession number AB199316) transcript levels. The expression levels are presented in heatmap using fold-change values transformed to log2 format by MeV4.9. The color scale and log2 values (fold-change values) are shown at the top of heatmap. Genes were clustered according to their expression profiles. **b** Hierarchical analysis of expression patterns of *SlbZIP* genes in different tissues and developmental stages. Tomato cultivars used in this analysis are MicroTom and Ailsa Craig. Different developmental stages and tissues used for analysis are shown and listed on the left. Group numbers were labelled after each gene name

To gain insight into the temporal and spatial transcriptional patterns and possible functions of *SlbZIP* genes in tomato growth and development, qRT-PCR analyses on 59 *SlbZIP* genes with EST support, together with publicly available microarray data sets for 26 *SlbZIPs*, were performed to examine the transcription levels in various tissues or organs, including the root, stem, leaf, flower, and fruit of plants. Transcriptional level for each of the 59 *SlbZIP* genes examined was detected in at least one of the tissues sampled (Fig. 6a). Notably, most of the *SlbZIP* genes analyzed showed overlapping expression patterns at least in two or more different tissues (Fig. 6a). Several *bZIP* genes did not show significant differences in their expression levels among different organs or tissues. In the analyzed 59 *SlbZIP* genes, most of the genes show high expression levels in roots whereas a small numbers of genes (*SlbZIP17*, *SlbZIP46*, *SlbZIP49*, *SlbZIP50* and *SlbZIP62*) presented very low expression in roots (Fig. 6a). Very high levels of expression were also detected for several genes (*SlbZIP01, SlbZIP013, SlbZIP16, SlbZIP36, SlbZIP38, SlbZIP45, SlbZIP50, SlbZIP51*, *SlbZIP53* and *SlbZIP61*) in fruit stages, among which *SlbZIP13 (LeGBF12)*, *SlbZIP16 (LeGBF4)* and *SlbZIP51 (LeGBF9)* have already been reported to express constitutively during fruit development [57]. These results were also supported by the microarray data obtained from GENEVESTIGATOR tool (Fig. 6b) [58]. Specifically, *SlbZIP49* and *SlbZIP50* exhibited same expression levels, which show very low expression potentials in root, stem, leave and flower and high expression potentials in fruit (Fig. 6a), while *SlbZIP02*, *SlbZIP 15*, *SlbZIP 24* and *SlbZIP 67* exhibit same expression potentials which show very low expression potentials in stem, leaf, flower and fruit but high expression potentials in root (Fig. 6a). Taken together, these data indicate that these selected *SlbZIP* genes have tissue-specific expression potentials in tomato. Notably, *SlbZIP61*, the duplicated gene of *SlbZIP02*, showed relatively high transcript abundance in root, leave and fruit, suggesting the difference expression pattern between duplicated gene pairs (Fig. 6a and b). Similar results were also found in other duplicated gene pairs (Fig. 6a and b). The divergences in expression profiles between orthologs revealed that some of them may acquire new functions after duplication in the evolutionary process.

### Expression patterns of the *SlbZIP* genes in response to phytohormones, abiotic stresses and in response to light

**Fig. 7:**
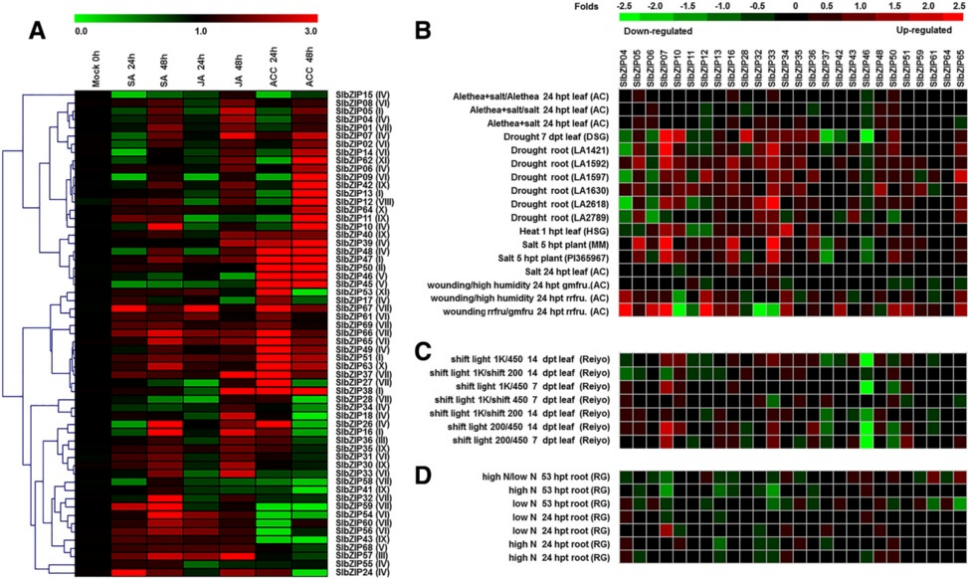
Expression patterns of the *SlbZIP* genes in response to phytohormone and abiotic stress treatments. **a** Expression patterns of *SlbZIP* genes under phytohormone treatments by qRT-PCR analysis. **b** Expression patterns of the *SlbZIP* genes in response to heat, salt, drought and wounding stress treatments. **c** Expression patterns of the *SlbZIP* genes in response to light shift. **d** Expression patterns of the *SlbZIP* genes in response to nitrogen stress. Group numbers were labelled after each gene name

Phytohormones such as SA, JA and ethylene act as endogenous messengers in plant response to biotic and abiotic stress [59]. It was reported that treatments of plants with exogenous hormones often result in transient and rapid genome-wide transcript changes [60]. We thus examined the responsiveness of most *SlbZIP* genes to exogenously applied SA, methyl jasmonate (MeJA) and 1-amino cyclopropane-1-carboxylic acid (ACC, a precursor of ethylene). Approximately 20 % of *SlbZIP* genes were up-regulated upon SA and JA treatment whereas about 45 % of *SlbZIP* genes were up-regulated upon ACC treatment (Fig. 7). The expression profiles of *SlbZIP* genes under SA and MeJA treatments showed similar expression patterns. As shown in Fig. 7a, ACC treatment resulted in great changes of expression levels for most of *SlbZIP* genes. These results indicate that the expression patterns of *SlbZIPs* can be regulated by different phytohormones.

We next analyzed the expression patterns of the 26 *SlbZIP* genes in tomato plants after treatments with other abiotic stresses using GENEINVESTIGATOR, a tool that allow to explore public expression data for specific genes, such as salt stress, drought stress, heat stress and nitrogen stress (Fig. 7b). The transcript levels of most tested bZIP genes also markedly varied during abiotic stress, implying their involvements in tomato abiotic stress response. Notably, expression of *SlbZIP10*, *SlbZIP32* and *SlbZIP33* in leaves and roots was significantly upregulated during drought, salt and heat stress conditions but was markedly downregulated in fruit tissue under wounding stress. This is consistent with previous reports that overexpression of *SlAREB* (*SlbZIP33*) improved plant tolerance to water deficit and salt stress and that *SlAREB* functions to regulate some stress-responsive genes (Fig. 7b) [24, 25]. By contrast, *SlbZIP04*, *SlbZIP06*, *SlbZIP37* and *SlbZIP46* in leaves and roots were significantly downregulated during drought, salt and heat stress conditions but were markedly upregulated in fruit tissue under wounding stress (Fig. 7b). These results were supported by the research on LebZIP2 (*SlbZIP04*), whose expression was strongly induced by NaCl and mannitol treatments [61]. Expression of other *SlbZIP* genes was affected by most of the stresses examined leading to upregulated or downregulated levels; however, Alethea [62] stress did not affect the expression of almost all of the selected *SlbZIP* genes (Fig. 7a). In particular, *SlbZIP07, SlbZIP13* and *SlbZIP33* in roots, *SlbZIP33, SlbZIP61* and *SlbZIP65* in root*,* significantly downregulated under high nitrogen and low nitrogen stresses, respectively (Fig. 7c).

Moreover, it was previously reported that some bZIP TFs regulate promoters of light-responsive genes. For example, the elongated HYPOCOTYL5 (HY5) bZIP protein, an integrator of multiple signaling pathways, plays an important role in photomorphogenic growth and light-regulated gene expression [63, 64]. To examine whether such a light-responsive relationship exists in the case of *SlbZIP* genes, we analyzed the expression patterns of the 26 *SlbZIP* genes under different light conditions (Fig. 7c). Notably, *SlbZIP46* in leaves was strongly downregulated in almost all the light shift conditions, e.g. from high light to low light and from low light to high light, while *SlbZIP07* was dramatically upregulated in all light shift conditions (Fig. 7c). Decreased expressions for *SlbZIP37* and *SlbZIP50* in all light shift conditions, *SlbZIP37* under light shift from 1000 to 450 μmol m^−2^ s^−1^, *SlbZIP34* under light shift from 450 to 1000 μmol m^−2^ s^−1^ and *SlbZIP04* and *SlbZIP07* under light shift from 1000 to 200 μmol m^−2^ s^−1^ were observed (Fig. 7c). These data indicate that a reasonable number of *SlbZIPs* display light-dependent expression patterns, which may be involved in some light-dependent biological processes.

### Expression of the *SlbZIP* genes in response to pathogens and elicitors

**Fig. 8:**
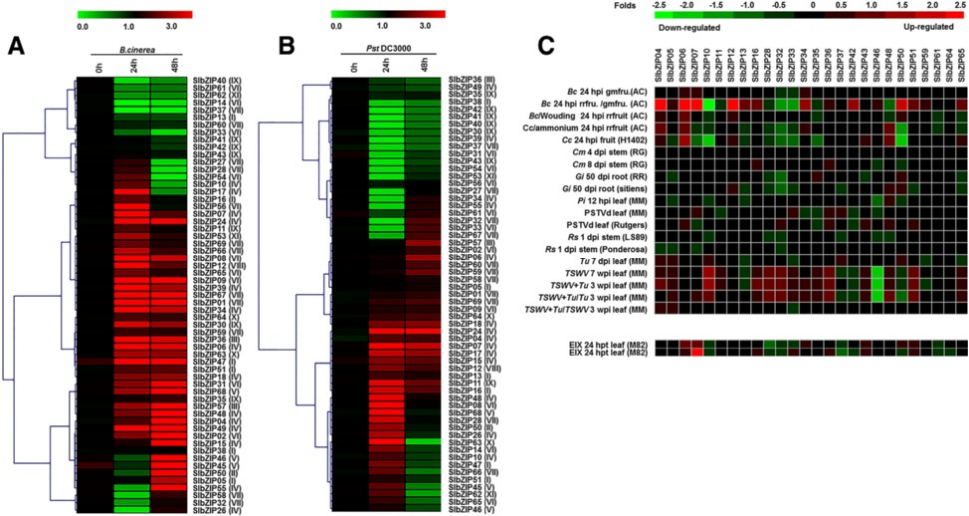
Expression patterns of the *SlbZIP* genes in response to pathogen infection and elicitor treatment. **a** qRT-PCR analysis of the expression patterns of *SlbZIP* genes in response to *B.cinerea*. **b** qRT-PCR analysis of the expression patterns of *SlbZIP* genes in response to *Pst* DC3000. **c** Expression patterns of *SlbZIP* genes in response to pathogen infection by public microarray data. Group numbers were labelled after each gene name

To explore the possible involvement of *SlbZIP* genes in defense response against pathogens, we analyzed the expression patterns of the selected 59 *SlbZIP* genes in tomato plants after infection with *Botrytis cinerea*, a necrotrophic fungal pathogen causing grey mold disease, or *Pseudomonas syringae* pv. *tomato* DC3000 (*Pst* DC3000), a (hemi) biotrophic bacterial pathogen causing bacterial leaf spot disease. As shown in Fig. 8, differential expression patterns of *SlbZIP* genes in response to pathogen infection were observed. Thirty two and fourteen *SlbZIP* genes were found to be up-regulated and down-regulated in response to *B. cinerea* and *Pst* DC3000 either 24 or 48 hpi or both, respectively (Fig. 8a, b). Most of the 32 up-regulated *SlbZIP* genes belong to group IV and VI, while most of the 14 down-regulated SlbZIP genes belong to group VI and VII. As shown in Fig. 8a, b, expression of 19 and 17 *SlbZIP* genes was also found to be downregulated and upregulated in response to *Pst* DC3000 after either 24 or 48 hpi or both, respectively. Most of the 19 down-regulated *SlbZIP* genes belong to group IX, VII and VI, while most of the 17 downregulated *SlbZIP* genes belong to IV group. Overall, 32 and 19 *SlbZIP* genes were up-regulated (>2-fold) after *B. cinerea* and *Pst* DC3000 infection as compared to the mock controls and 14 and 17 *SlbZIP* genes were down-regulated after *B. cinerea* and *Pst* DC3000 infection, respectively.

We also examined the expression patterns of the 26 *SlbZIP* genes in tomato after infection with other pathogens or treatments with some well-known effectors or elicitors from pathogenic fungi using Genvestigator tool and observed differential expression patterns (Fig. 8c). Expression of *SlbZIP10* and *SlbZIP50* in fruits infected with *C. coccodes* and expression of *SlbZIP46* in leaves infected with *Tomato Spotted Wilt Virus* (*TSWV*) or *TSWV+ Tetranychus urticae* (*Tu*) were significantly reduced (Fig. 8c). Infection by *Glomus intraradices* (*Gi*), *Phytophthora infestans* (*Pi*), *Ralstonia solanacearum* (*Rs*) or *Potato Spindle Tuber Viroid* also resulted in changes of expression levels for some of *SlbZIP* genes (Fig. 8c). Notably, expression of *SlbZIP07* was significantly upregulated after treatment with EIX, a protein elicitor isolated from fungus *Trichoderma viride* [65]. These results imply that a number of the *SlbZIP* genes show differentially expression patterns upon infection by different pathogens including fungi, oomycetes and viruses and pathogen-derived effectors or elicitors.

### Five selected SlbZIPs are localized in nucleus and have transactivation activity

**Fig. 9:**
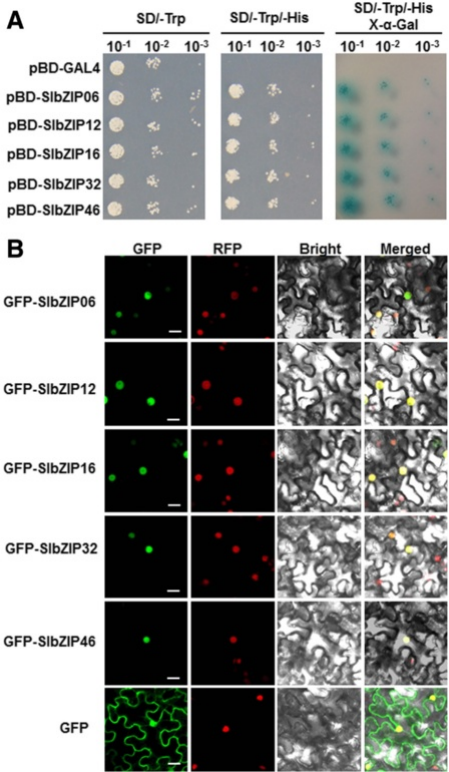
Analysis of transactivation activities and subcellular localization of SlbZIP6, SlbZIP12, SlbZIP16, SlbZIP32 and SlbZIP46. **a** Transactivation activity of SlbZIP6, SlbZIP12, SlbZIP16, SlbZIP32 and SlbZIP46 in yeast. Yeasts carrying pBD-SlbZIP6/12/16/32/46 or pBD empty vector (as a negative control) were streaked on the SD/Trp^−^ plates (left) or SD/Trp^−^ His^−^ plates (middle) or SD/Trp^−^His^−^/X-α-Gal plates (right) for 3 days at 30 °C. **b** Subcellular localization of SlbZIP6, SlbZIP12, SlbZIP16, SlbZIP32 and SlbZIP46. Agrobacteria carrying SlbZIP6, SlbZIP12, SlbZIP16, SlbZIP32, SlbZIP46 or control vector GFP were infiltrated into leaves of *N. benthamiana* plants and the fluorescence images were taken in dark field for green fluorescence (left), in dark field for red fluorescence (nuclear maker) (middle left), in white field for the morphology of the cell (middle right), and in combination (right), respectively. Scale bar = 20 μm

To gain an insight into the biochemical characteristics and subcellular localization, five SlbZIPs including SlbZIP06, SlbZIP12, SlbZIP16, SlbZIP32 and SlbZIP46, representing different groups, were selected to examine their transactivation activity in yeast and subcellular localization *in planta*. Firstly, we examined whether these selected SlbZIP proteins had transactivation activity using a yeast assay system. As shown in Fig. 9a, all yeast transformants grew well on SD/Trp^−^ medium. However, only yeast transformants containing pBD-SlZIP06, pBD-SlbZIP12, pBD-SlbZIP16, pBD-SlbZIP32 or pBD-SlbZIP46 were able to grow on the SD/Trp^−^His^−^ medium and produced a blue pigment after the addition of x-α-gal, showing β-galactosidase activities, whereas transformants containing the pBD empty vector did not. These results indicate that all these five SlbZIP proteins have transactivation activity in yeasts. Furthermore, we also examined their subcellular localizations using a transient expression approach. We transiently expressed GFP-tagged SlbZIP06, SlbZIP12, SlbZIP16, SlbZIP32 or SlbZIP46SlSR1 in leaves of 4-week-old *N. benthamiana* plants that express a red nuclear marker protein RFP–H2B [66] by infiltration with agrobacteria carrying pFGC-Egfp-SlbZIP06, pFGC-Egfp-SlbZIP12, pFGC-Egfp-SlbZIP16, pFGC-Egfp-SlbZIP32, pFGC-Egfp-SlbZIP46, or pFGC-Egfp constructs and GFP was observed at 2 days after agroinfiltration. As shown in Fig. 9b, the GFP-SlbZIP06, GFP-SlbZIP12, GFP-SlbZIP16, GFP-SlbZIP32 and GFP-SlbZIP46 fusions accumulated exclusively in the nuclei nucleus of *N. benthamiana* cells, co-localized with the known nucleus marker RFP–H2B, whereas the GFP protein alone accumulated in both the cytoplasm and the nucleus, demonstrating that all the SlbZIP06, SlbZIP12, SlbZIP16, SlbZIP32 and SlbZIP46 proteins are localized in the nucleus of cells. Taken together, our experimental data demonstrate that SlbZIP06, SlbZIP12, SlbZIP16, SlbZIP32 and SlbZIP46 are nucleus-localized transcriptional activators. These observations were also consistent with previous reports [67–70].

It was reported that some bZIP proteins can form hetero- or homodimers to function cooperatively and that SA is required for full activation of these bZIP proteins in tomato and Arabidopsis [30, 71]. The expression of *SlbZIP06*, *SlbZIP12*, *SlbZIP16*, *SlbZIP32* and *SlbZIP*46 was found to be regulated by phytohormones such as SA, JA or ACC (Fig. 7), indicating that these five *SlbZIP* genes may have function in phytohormone-mediated biological processes, probably through the hetero- or homodimerization in vivo.

## Conclusion

bZIP TFs have been characterized in different plant species and implicated in various critical developmental and physiological processes. However, only a few tomato *SlbZIP* genes have been studied so far for their biological functions and the information on the tomato SlbZIP family is also lacking. In the present study, we performed a genome-wide systematic characterization of the tomato SlbZIP family and a total of 69 SlbZIPs were identified. Importantly, an extensive characterization of the SlbZIPs was performed in terms of the gene structures, chromosomal distribution and evolution, the conserved amino acid residues within bZIP domain, the conserved motifs, DNA-binding site specificity and dimerization property, phylogenetic relationships and expression patterns among different tissues and in response to abiotic and biotic stress as well as in response to light. Furthermore, 5 selected SlbZIPs were characterized biochemically for their subcellular localization and transactivation activity. This genome-wide systematic characterization of the tomato SlbZIP family provides a useful platform for further functional studies of SlbZIPs in tomato.

## Methods

### Plant growth conditions and treatments

Tomato (*Solanum lycopersicum*) cv. Suhong 2003 was used for all gene expression analysis by qRT-PCR. Seedlings were grown a mixture of perlite: vermiculite: plant ash (1:6:2) in a growth room under fluorescent light (200 μE m^2^ s^−1^) at 22–24 °C with 60 % relative humidity and a 14 h light/10 h dark cycle. For analysis of gene expression, 4-week-old tomato plants were treated by foliar spraying with 100 μM MeJA, 100 μM ACC, 100 μM SA or 0.1 % ethanol as a control and samples were collected at indicated time points after treatment. Pathogen inoculation with *B. cinerea* or with *Pst* DC3000 was performed basically according to previously described protocols [72]. Leaf samples were collected at indicated time points after treatment or inoculation and stored at - 80 °C until use.

### Identification of bZIPs in tomato

Both local BLAST and hidden Markov model profile searches against the tomato genome database at the Sol Genomics Network (SGN; http://solgenomics.net/) using Arabidopsis and rice bZIP protein sequences, which were downloaded from The Arabidopsis Information Resource (http://www.arabidopsis.org/) and Rice Genome Annotation Project (http://rice.plantbiology.msu.edu/) databases, respectively, as queries. The *E*-value for searches was set to 1. The obtained sequences were subjected to further searching at the National Center of Biotechnology Information CD search (http://www.ncbi.nlm.nih.gov/Structure/cdd/wrpsb.cgi), SMART (http://smart.embl-heidelberg.de/), PROSITE (http://www.expasy.org/prosite/), and Pfam (http://pfam.sanger.ac.uk/) databases for the presence of the bZIP domain. After removing the repeat and incomplete sequences manually, the remaining sequences were considered as candidates of SlbZIPs and subjected to further analyses.

### Phylogenetic tree analyses

Multiple sequence alignments of the SlbZIP full proteins, basic regions and Leu zipper domains were performed using ClustalX (version 2.0.8) followed by manual adjustment. Phylogenetic trees generated by the neighbor-joining (NJ) algorithm with p-distance method and pairwise deletion of gaps using MEGA version 6.06 with default parameters [73]. A bootstrap statistical analysis was performed with 1000 replicates to test the phylogeny.

### Identification of additional conserved motifs in SlbZIPs

The SlbZIP protein sequences were submitted to Multiple Em (Expectation Maximization) for the Motif Elicitation tool (MEME version 4.9.1, http://meme.sdsc.edu/meme/cgi-bin/meme.cgi). The limits of minimum width, maximum width and maximum number of motifs were specified as 10, 50 and 50, respectively to exclude the bZIP domain. The motifs with low *E*-value (<*E* − 48) were finally confirmed, numbered according to their order displayed in MEME and considered as group-specific signatures for their presence of high frequency in the given groups.

### Analyses of gene structure and conserved intron splicing site

Both *SlbZIP* gene sequences and corresponding coding sequences were loaded into the Gene Structure Display Server (http://gsds.cbi.pku.edu.cn/) [74]. The 5′ UTR sequences of each gene were removed for a better visualization and comparison. The cDNA sequences were aligned with their corresponding genomic sequences using Spidey (http://www.ncbi.nlm.nih.gov/spidey/) to obtain the intron/exon structure for each gene. Information on intron distribution pattern and intron splicing phase within the basic and hinge regions of the bZIP domains were derived from the aligned cDNA sequences.

### Chromosomal distribution and detection of duplication events

The *SlbZIP* genes were mapped onto the corresponding chromosomes by identifying their chromosomal positions provided in the Sol Genomics Network (SGN; http://solgenomics.net/). The syntenic blocks used for constructing a synteny analysis map of the *SlbZIP* genes were obtained from the Plant Genome Duplication Database [75] and the diagrams were generated by the program Circos version 0.63 (http://circos.ca/) [76].

### Gene expression analyses

Total RNA was extracted using TRIzol reagent (Invitrogen, Shanghai, China) and treated with RNase-free DNase (TaKaRa, Dalian, China) according to the manufactures’ instructions. For qRT-PCR analysis, RNA samples were reverse transcribed with oligo(dT) using PrimeScript reagent kit with gDNA eraser (TaKaRa, Dalian, China). qRT-PCR was performed on a CFX96 Real-Time PCR detection system (BioRad, Hercules, CA, USA) using SYBR Premix Ex TaqTM kits (TaKaRa, Dalian, China). Tomato *Actin1* gene (*SlActin*) was used as the internal standard for normalizing the qRT-PCR data. Three independent biological replicates were done. Relative expression levels were calculated using the 2^-ΔΔCT^ method. Gene-specific primers for the *SlbZIP* genes were designed according to the predicted mRNA sequence. The sequences of primers and their products were listed in Additional file 12: Table S7. To visualizing the relative fold difference, all data were normalized based on setting up the relative expression level, the expression level of 0-point treatments for phytohormone and pathogen infection was set as 1. The qRT-PCR data were clustered with Pearson correlation distance metric using the average linkage method by MeV 4.9 software (http://www.tm4.org) [77].

Microarray expression data from various datasets were obtained using Genevestigator (https://www.genevestigator.com/gv/) with the tomato Gene Chip platform. The web site provides a web-based search interface to search for probes of genes on the tomato GeneChip by using keywords as well as probe ID numbers and GO terms as query terms. The expression data for each gene in different development stages, organs and under different abiotic and biotic stress conditions were mined. Results are given as heatmaps in different color coding that reflects absolute signal values. The color scale with heatmap is given in log_2_ ratio values. Tomato cultivars used in the microarray analysis were: AC, cv. Ailsa Craig; RG, cv. Rio Grande; RR, cv. Rheinlands Ruhm; MM, cv. MoneyMaker; DSG, Drought Susceptible Genotype; HSG, Heat Susceptible Genotype. The tomato microarray expression analyses were obtained from samples infected by *B. cinerea* (*Bc*), *C. coccodes* (*Cc*), *Clavibacter michiganensis* (*Cm*), *P. infestans* (*Pi*), *Potato Spindle Tuber Viroid* (*PSTVd*), *R. solanacearum* (*Rs*), *T. urticae* (*Tu*), *Tomato Spotted Wilt Virus* (*TSWV*), treated with elicitor EIX (ethylene-inducing xylanase, 2.5 μg/ml) or colonized by *G. intraradices* (*Gi*). Abiotic stress treatments in tomato microarray expression analyses include ‘Alethea’ (1:99 v/v), drought (withholding water), heat (40 °C), salt (NaCl, 200 mM), or wounding (puncturing a disinfected fruit). Time points of sampling are indicated as dpt (days post treatment), hpt (hours post treatment), dpi (days post inoculation) or hpi (hours post inoculation) and tissues sampled for analyses are also indicated as root, leaf, gmfru (green mature fruit) or rrfru (red ripe fruit).

### Transactivation activity and subcellular localization assays

For transactivation activity assays, the entire coding sequences of *SlbZIP06, SlbZIP12, SlbZIP16, SlbZIP32 and SlbZIP46* were amplified using gene-specific primers (Additional file 12: Table S7) and fused in frame to the yeast GAL4 DNA binding domain in vector pBD-GAL4Cam with corresponding restriction enzymes, yielding plasmid pBD-SlbZIP06, pBD-SlbZIP12, pBD-SlbZIP16, pBD-SlbZIP32 and pBD-SlbZIP46, respectively. These plasmids and pBD empty vector (negative control) were transformed into yeast strain AH109. The transformed yeast strains were plated on SD/-Trp medium or SD/-Trp-His medium and cultivated for 3 days at 30 °C, followed by addition of X-α-Gal. Transactivation activity of the fused proteins was evaluated according to the growth situation and production of blue pigments after the addition of X-α-Gal of the transformed yeast cells on the SD/-Trp-His medium.

For subcellular localization assays, the entire coding sequences of *SlbZIP06, SlbZIP12, SlbZIP16, SlbZIP32 and SlbZIP46* were amplified using gene-specific primers (Additional file 12: Table S7) and inserted into vector pFGC-EGFP after digestion with *Bam*HI and *Xba*I, yielding plasmid pFGC-GFP-bZIP06, pFGC-GFP-bZIP12, pFGC-GFP-bZIP16, pFGC-GFP-bZIP32 and pFGC-GFP-bZIP46, respectively. This plasmid and the pFGC-EGFP empty vector were transformed into *Agrobacterium tumefacies* GV3101 and the transformed agrobacteria were infiltrated individually into leaves of 4-week-old *N. benthamiana* plants expressing a red nuclear marker RFP-Histone2B protein [66] using 1-ml needless syringes. After agroinfiltration, the plants were grown in a growth room under 25 °C for 48 h. GFP fluorescence signals were excited at 488 nm and detected using a 500–530 nm emission filter preformed with Zeiss LSM 780 confocal laser scanning microscope (Carl Zeiss, Germany).

## Availability of supporting data

The data sets supporting the results of qRT-PCR assays are available in the Gene Expression Omnibus (GEO) repository under accession numbers of GSE72189 (http://www.ncbi.nlm.nih.gov/geo/query/acc.cgi?acc=GSE72189) and GSE72215 (http://www.ncbi.nlm.nih.gov/geo/query/acc.cgi?acc=GSE72215). Sequence information of plant *bZIP* genes used in phylogenetic trees was deposited in the LabArchives under the DOI ‘10.6070/H4WD3XKK’ (https://mynotebook.labarchives.com/share/Dayong%2520Li/MjAuOHwxMDIwMDkvMTYvVHJlZU5vZGUvMzMyNTU0MzA3Mnw1Mi44).

## Additional files

Additional file 1: Table S1. The identified tomato bZIP proteins and their related information. (DOC 166 kb) Additional file 2: Figure S1. Phylogenetic relationship among the SlbZIP proteins based on (a) bZIP domain (b) basic and hinge regions (c) leucine zipper defined from the first leucine. The unrooted tree was generated using neighbor-joining method by MEGA6.06. Bootstrap values from 1000 replicates are indicated at each node. (JPEG 12623 kb) Additional file 3: Figure S2. Phylogenetic relationship among the tomato, Arabidopsis and rice bZIP proteins. The unrooted tree was generated using neighbor-joining method by MEGA6.06. Bootstrap values from 1000 replicates are indicated at each node. Protein names of already characterized bZIP proteins have been indicated. (JPEG 8739 kb) Additional file 4: Table S2. The additional conserved motifs of SlbZIP proteins in each group as predicted by MEME. (DOC 87 kb) Additional file 5: Table S3. DNA binding specificity prediction of SlbZIP transcription factors for each group. (DOCX 19 kb) Additional file 6: Figure S3. Amino acid sequence alignment of the leucine zipper region of 69 SlbZIP proteins. The boundaries of the Leu zippers in SlbZIPs were defined according to the criteria used for the *Arabidopsis*, rice and maize bZIP proteins and the Leu zipper regions were then arranged in the form of heptad repeats, in which the amino acid positions of each heptad was named *g*, *a*, *b*, *c*, *d*, *e* and *f* in order. Four colors are used to differentiate between different *g*↔*e*’pairs. Attractive basic-acidic pairs (R↔E and K↔E) are colored orange, attractive acidic-basic pairs (E↔R, E↔K, D↔R, and D↔K) are blue, repulsive basic pairs (K↔K, R↔K, R↔Q, Q↔K, and K↔Q) are purple and repulsive acidic pairs (E↔E, E↔D, E↔Q, and Q↔E) are green. If only one of the two amino acids in the *g*↔*e*’ pair is charged, the residue is colored purple for basic and green for acidic. If the *a* or *d* position is charged, it is colored brown. Asparagines at *a* position are colored red. The prolines and glycines are bold to indicate a potential break in the α-helix. The predicted C-terminal boundary is denoted by the symbol **#**. (TIFF 14383 kb) Additional file 7: Table S4. Classification of SlbZIP proteins into sub-families with similar predicted dimerization specificity. (DOCX 23 kb) Additional file 8: Figure S4. The map of intron-exon arrangement of *SlbZIP* genes. (JPEG 24649 kb) Additional file 9: Figure S5. Position and pattern of introns within the basic and hinge regions of the bZIP domains of the SlbZIP transcription factors. (JPEG 11880 kb) Additional file 10: Table S5. Gene clusters of SlbZIP transcription factor family. (DOC 50 kb) Additional file 11: Table S6. SlbZIP genes present on duplicated chromosomal segments. (DOC 70 kb) Additional file 12: Table S7. Primers used in this study. (DOC 132 kb)

## Abbreviations

ACC: 1-amino cyclopropane-1-carboxylic acid
*B. cinerea*: *Botrytis cinerea*
bZIP: Basic leucine zipper
EST: Expressed sequence tag
GFP: Green fluorescent protein
MeJA: Methyl jasmonate
MEME: Multiple expectation maximization for motif elicitation
ORF: Open reading frame
*Pst* DC3000: *Pseudomonas syringae* pv. *tomato*
qRT-PCR: Quantitative reverse transcription polymerase chain reaction
SA: Salicylic acid
TFs: Transcription factors
