# Supplementary material for: Genome-wide systematic characterization of the bZIP transcriptional factor family in tomato (Solanum lycopersicum L.)
Source: BMC Genomics. 2015 Oct 12;16:771. doi: 10.1186/s12864-015-1990-6 (PMC4603586; doi:10.1186/s12864-015-1990-6)
Supplement: Additional file 1: Table S1. — The identified tomato bZIP proteins and their related information. (DOC 166 kb) [file 12864_2015_1990_MOESM1_ESM.doc]

Additional file 1: Table S1. Information on the tomato SlbZIP family

| ***SlbZIP*** | **SOL Locus ID** | **Group** | **Gene feature** | | | **Protein feature** | | | | **Previous**  **names** | **cDNAs**  **(NCBI/SGN)** |
| --- | --- | --- | --- | --- | --- | --- | --- | --- | --- | --- | --- |
|  |  |  | **Chr.** | **No. of**  **intron** | **Intron pattern within the basic and hinge region of the bZIP domain** | **Size**  **(aa)** | **Position of**  **bZIP domain** | **MW**  **(kD)** | ***p*I** |  |  |
| ***SlbZIP01*** | Solyc01g008730 | VII | 1 | 7 | *c* | 327 | 39-102 | 36.37 | 8.59 |  | SGN-U586165 |
| ***SlbZIP02*** | Solyc01g008980 | VI | 1 | 2 | *b* | 146 | 75-134 | 16.69 | 9.22 |  | SGN-U603508 |
| ***SlbZIP03*** | Solyc01g009510 | III | 1 | 3 | *b* | 190 | 107-172 | 21.31 | 6.85 |  | / |
| ***SlbZIP04*** | Solyc01g079480 | IV | 1 | 0 | *f* | 144 | 19-83 | 16.60 | 6.14 | LebZIP2 | AK32888/SGN-U568869 |
| ***SlbZIP05*** | Solyc01g095460 | I | 1 | 11 | *b* | 418 | 275-339 | 44.66 | 5.73 |  | AK325748/SGN-U570651 |
| ***SlbZIP06*** | Solyc01g097330 | IV | 1 | 5 | *a* | 454 | 247-311 | 48.61 | 6.24 |  | SGN-U572086 |
| ***SlbZIP07*** | Solyc01g100460 | IV | 1 | 0 | *f* | 138 | 21-85 | 15.42 | 9.55 | ABZ1 | AK328435/SGN-U576188 |
| ***SlbZIP08*** | Solyc01g104650 | VI | 1 | 3 | *b* | 297 | 213-277 | 32.08 | 7.81 |  | AK247103/SGN-U578362 |
| ***SlbZIP09*** | Solyc01g108080 | VI | 1 | 3 | *b* | 414 | 332-393 | 45.03 | 9.64 |  | SGN-U284254 |
| ***SlbZIP10*** | Solyc01g109880 | IV | 1 | 0 | *f* | 163 | 26-90 | 18.66 | 5.91 | LebZIP1 | AK327140/SGN-U566266 |
| ***SlbZIP11*** | Solyc01g110480 | IX | 1 | 3 | *a* | 582 | 417-481 | 63.70 | 6.83 |  | BT014576/SGN-U579948 |
| ***SlbZIP12*** | Solyc01g111580 | VIII | 1 | 1 | *f* | 270 | 83-150 | 29.48 | 6.15 |  | AK327110/SGN-U567970 |
| ***SlbZIP13*** | Solyc02g062950 | I | 2 | 10 | *b* | 341 | 242-306 | 36.59 | 5.67 | LeGBF12 | AK324421/SGN-U574397 |
| ***SlbZIP14*** | Solyc02g083520 | VI | 2 | 2 | *b* | 217 | 144-208 | 24.27 | 9.51 |  | SGN-U564495 |
| ***SlbZIP15*** | Solyc02g084860 | IV | 2 | 0 | *f* | 144 | 18-82 | 16.43 | 5.38 |  | SGN-U571725 |
| ***SlbZIP16*** | Solyc02g085610 | I | 2 | 7 | *b* | 284 | 186-250 | 30.79 | 5.52 | LeGBF4 | X74942/ SGN-U574396 |
| ***SlbZIP17*** | Solyc02g089420 | IV | 2 | 0 | *f* | 180 | 77-141 | 21.05 | 5.95 |  | AK321500 |
| ***SlbZIP18*** | Solyc02g092090 | IV | 2 | 0 | *f* | 214 | 110-174 | 24.82 | 9.95 |  | SGN-U568565 |
| ***SlbZIP19*** | Solyc03g033730 | IV | 3 | 0 | *f* | 166 | 16-80 | 13.40 | 9.77 |  | / |
| ***SlbZIP20*** | Solyc03g043810 | IV | 3 | 2 | *a* | 124 | 48-121 | 14.39 | 10.14 |  | / |
| ***SlbZIP21*** | Solyc03g043820 | IV | 3 | 3 | *a* | 126 | 46-110 | 14.83 | 9.68 |  | / |
| ***SlbZIP22*** | Solyc03g043830 | IV | 3 | 3 | *a* | 174 | 48-119 | 20.53 | 5.31 |  | / |
| ***SlbZIP23*** | Solyc03g043840 | IV | 3 | 3 | *a* | 170 | 48-119 | 20.10 | 5.19 |  | / |
| ***SlbZIP24*** | Solyc03g043900 | IV | 3 | 0 | *f* | 203 | 82-146 | 23.65 | 9.39 |  | SGN-U591210 |
| ***SlbZIP25*** | Solyc03g046440 | IV | 3 | 1 | *f* | 173 | 66-130 | 20.28 | 6.16 |  | / |
| ***SlbZIP26*** | Solyc04g005170 | IV | 4 | 0 | *f* | 191 | 77-141 | 22.30 | 5.98 |  | AK329122/SGN-U572908 |
| ***SlbZIP27*** | Solyc04g011670 | VII | 4 | 7 | *c* | 374 | 84-144 | 42.40 | 6.11 |  | AK319929/SGN-U570984 |
| ***SlbZIP28*** | Solyc04g054320 | VII | 4 | 7 | *c* | 370 | 82-145 | 41.76 | 5.37 |  | AK320487/SGN-U570983 |
| ***SlbZIP29*** | Solyc04g056460 | III | 4 | 1 | *f* | 490 | 2-59 | 53.92 | 9.43 |  | / |
| ***SlbZIP30*** | Solyc04g071160 | IX | 4 | 3 | *a* | 341 | 180-244 | 37.52 | 6.61 |  | AK323544/SGN-U577485 |
| ***SlbZIP31*** | Solyc04g071510 | VI | 4 | 3 | *b* | 308 | 224-286 | 33.87 | 6.71 |  | SGN-U576153 |
| ***SlbZIP32*** | Solyc04g072460 | VII | 4 | 7 | *c* | 361 | 75-135 | 40.88 | 6.72 |  | BT014216/SGN-U570985 |
| ***SlbZIP33*** | Solyc04g078840 | VI | 4 | 3 | *b* | 447 | 365-430 | 47.98 | 9.42 | *SlAREB1* | AY530758/ SGN-U565963 |
| ***SlbZIP34*** | Solyc04g080740 | IV | 4 | 0 | *f* | 147 | 23-87 | 17.27 | 7.82 |  | AK322965/SGN-U566267 |
| ***SlbZIP35*** | Solyc04g081190 | IX | 4 | 3 | *a* | 444 | 295-359 | 48.28 | 8.98 | *VSF-1* | AK247799/SGN-U564727 |
| ***SlbZIP36*** | Solyc04g082890 | III | 4 | 2 | *a* | 284 | 120-184 | 32.22 | 5.65 |  | AK224603/ SGN-U580933 |
| ***SlbZIP37*** | Solyc05g009660 | VII | 5 | 10 | *c* | 484 | 192-257 | 53.30 | 6.13 |  | SGN-U564702 |
| ***SlbZIP38*** | Solyc05g050220 | I | 5 | 11 | *b* | 416 | 273-337 | 44.92 | 8.86 |  | SGN-U273679 |
| ***SlbZIP39*** | Solyc06g009640 | IV | 6 | 0 | *f* | 144 | 20-84 | 16.62 | 5.51 |  | AK326780/SGN-U567159 |
| ***SlbZIP40*** | Solyc06g048630 | IX | 6 | 3 | *a* | 349 | 146-210 | 38.64 | 5.9 |  | AK32261/SGN-U585731 |
| ***SlbZIP41*** | Solyc06g049040 | IX | 6 | 3 | *a* | 340 | 136-200 | 36.71 | 6.02 |  | SGN-U565193 |
| ***SlbZIP42*** | Solyc06g053350 | IX | 6 | 3 | *a* | 434 | 222-286 | 47.52 | 5.97 |  | AK247599/SGN-U584071 |
| ***SlbZIP43*** | Solyc06g060490 | IX | 6 | 3 | *a* | 277 | 167-231 | 30.86 | 6.33 |  | AK324007/SGN-U579936 |
| ***SlbZIP44*** | Solyc06g074320 | VII | 6 | 11 | *c* | 501 | 192-271 | 55.60 | 6.53 |  | / |
| ***SlbZIP45*** | Solyc07g053450 | V | 7 | 3 | *a* | 284 | 176-240 | 32.72 | 5.82 |  | AK320822/SGN-U566274 |
| ***SlbZIP46*** | Solyc07g062710 | V | 7 | 3 | *a* | 302 | 178-242 | 33.59 | 5.69 |  | SGN-U578850 |
| ***SlbZIP47*** | Solyc08g005290 | I | 8 | 11 | *b* | 397 | 301-365 | 42.07 | 6.65 |  | AK320535/SGN-U431229 |
| ***SlbZIP48*** | Solyc08g006110 | IV | 8 | 5 | *a* | 351 | 178-242 | 38.86 | 5.60 |  | AK329008/SGN-U569399 |
| ***SlbZIP49*** | Solyc08g022080 | IV | 8 | 5 | *a* | 416 | 212-276 | 45.42 | 5.52 |  | AK327032/SGN-U572089 |
| ***SlbZIP50*** | Solyc08g061130 | II | 8 | 3 | *a* | 158 | 84-148 | 17.34 | 9.69 |  | AK329779/SGN-U568798 |
| ***SlbZIP51*** | Solyc08g076100 | I | 8 | 11 | *b* | 409 | 305-369 | 43.57 | 6.88 | LeGBF9 | AK322662/SGN-U583691 |
| ***SlbZIP52*** | Solyc09g009490 | VI | 9 | 3 | *b* | 426 | 338-402 | 46.07 | 8.79 |  | / |
| ***SlbZIP53*** | Solyc09g009760 | XI | 9 | 8 | *b* | 499 | 173-237 | 55.63 | 5.98 |  | SGN-U291461 |
| ***SlbZIP54*** | Solyc10g050210 | VI | 10 | 3 | *b* | 388 | 306-371 | 42.42 | 9.86 |  | SGN-U447654 |
| ***SlbZIP55*** | Solyc10g054010 | IV | 10 | 0 | *f* | 163 | 32-96 | 18.70 | 5.21 |  | SGN-U578320 |
| ***SlbZIP56*** | Solyc10g076920 | VI | 10 | 2 | *b* | 324 | 250-318 | 36.28 | 6.41 |  | SGN-U565413 |
| ***SlbZIP57*** | Solyc10g078290 | III | 10 | 1 | *f* | 660 | 175-239 | 70.98 | 9.23 |  | AK319272/SGN-U572181 |
| ***SlbZIP58*** | Solyc10g078670 | VII | 10 | 10 | *c* | 487 | 191-269 | 55.52 | 6.27 |  | SGN-U593980 |
| ***SlbZIP59*** | Solyc10g080410 | VII | 10 | 10 | *c* | 459 | 161-221 | 52.12 | 6.56 |  | SGN-U574124 |
| ***SlbZIP60*** | Solyc10g080770 | VII | 10 | 10 | *c* | 450 | 160-223 | 49.58 | 7.88 |  | AK325768/SGN-U571820 |
| ***SlbZIP61*** | Solyc10g081350 | VI | 10 | 2 | *b* | 350 | 276-348 | 38.40 | 8.63 |  | SGN-U283737 |
| ***SlbZIP62*** | Solyc10g083380 | XI | 10 | 4 | *b* | 299 | 2-59 | 33.75 | 9.24 |  | SGN-U574178 |
| ***SlbZIP63*** | Solyc11g006490 | X | 11 | 3 | *d* | 307 | 173-236 | 34.16 | 7.05 |  | SGN-U571171 |
| ***SlbZIP64*** | Solyc11g020950 | X | 11 | 4 | *e* | 326 | 177-236 | 37.19 | 7.31 |  | SGN-U572343 |
| ***SlbZIP65*** | Solyc11g044560 | VI | 11 | 5 | *b* | 365 | 283-348 | 44.14 | 8.83 | *SlAREB2* | AK325753/SGN-U571050 |
| ***SlbZIP66*** | Solyc11g064950 | VII | 11 | 7 | *c* | 324 | 36-99 | 36.17 | 8.86 |  | AK324242/SGN-U562604 |
| ***SlbZIP67*** | Solyc11g068370 | VII | 11 | 9 | *c* | 372 | 66-142 | 41.74 | 8.3 |  | SGN-U426050 |
| ***SlbZIP68*** | Solyc12g010800 | V | 12 | 3 | *a* | 306 | 175-239 | 34.99 | 5.47 |  | SGN-U570800 |
| ***SlbZIP69*** | Solyc12g056860 | VII | 12 | 7 | *c* | 348 | 62-125 | 39.56 | 6.62 |  | AK323598/SGN-U566338 |
