# Supplementary material for: Genome-wide systematic characterization of the bZIP transcriptional factor family in tomato (Solanum lycopersicum L.)
Source: BMC Genomics. 2015 Oct 12;16:771. doi: 10.1186/s12864-015-1990-6 (PMC4603586; doi:10.1186/s12864-015-1990-6)
Supplement: Additional file 4: Table S2. — The additional conserved motifs of SlbZIP proteins in each group as predicted by MEME. (DOC 87 kb) [file 12864_2015_1990_MOESM4_ESM.doc]

| **Additional file 4: Table S2.** The additional conserved motifs of SlbZIP proteins in each group as predicted by MEME. | | | | | | | |
| --- | --- | --- | --- | --- | --- | --- | --- |
| **Group** |  | **Motif No.** | **Length (aa)** | **E-value** | **Multilevel Consensus Sequence** | **No. of SlbZIP proteins in which motif is present** | **SlbZIP proteins in which motif is present** |
| I | I | 11 | 30 | 8.1e-041 | [GPT][PY][PG][HTV]PY[APV]A[LM]Y[PA][HP][GA]G[VI]YAHP[GNS][IV][PA][IP]GSYPFS[PH] | 6 | SlbZIP5,13,16,38,47,51 |
|  | I | 18 | 16 | 3.0e-019 | [MVT][AV][PSG][SG][PH][QTA][PA]HPYMWG[PVA][QL][HPQ] | 5 | SlbZIP5,13,38,47,51 |
|  | I | 20 | 31 | 2.6e-017 | [GHT][AG][GN][GP][IV][PAT][GM]P[AT][TP]NLNIGMD[YLV]W[NG]A[APS][AST][SAG][GPS][PNS][GI][PAM][AI][KIM][HIM] | 4 | SlbZIP,13,16,47,51 |
|  | I | 25 | 50 | 4.5e-013 | [NV][PQ][ED]KD[GS][KG][GS][PS]EG[KI][DEP]K[LN]P[AIS]K[KQR][KLS]K[GP][CGS][LS][GQ][GSV][KL][ANS][GMT][IT][ST]G[KS][NS]T[ES][GL][SG][GK][DNT][DS][GE]A[ES][AG][EN][AG][EV][TY][ST] | 5 | SlbZIP5,13,16,47,51 |
| II | II | 6 | 16 | 1.6e-122 | QL[EQ][AE]ENxRL[RKS]AQ[LG]xEL | 1 | SlbZIP50 |
| III | III | 6 | 16 | 1.6e-122 | QL[EQ][AE]ENxRL[RKS]AQ[LG]xEL | 4 | SlbZIP03,29,36,57 |
| IV | IV | 6 | 16 | 1.6e-122 | QL[EQ][AE]ENxRL[RKS]AQ[LG]xEL | 13 | SlbZIP04,06,07,10,15,17,18,19,23,24,25,26,34,39,48,49,55 |
|  | IV | 12 | 31 | 2.2e-030 | [IT][EP][YF]ES[VI]N[RKN]I[PS][ST]LEEA[LV]N[SL]E[IL][KE][HN]GQVDPN[MV]NM | 4 | SlbZIP20,21,22,23 |
|  | IV | 17 | 19 | 2.4e-023 | D[DT][LF]L[NE]PW[NQ]L[LP]C[VAP][NI]QPI[MT]AS | 9 | SlbZIP04,06,07,10,15,17,18,19,23,24,34,39,48,49 |
|  | IV | 22 | 22 | 7.7e-014 | KDLEE[RM]IAF[LF]GRKIENDKDNNK | 4 | SlbZIP20,21,22,23 |
|  | IV | 28 | 22 | 3.8e-006 | [NST][QN]LK[KE][EQ]NN[QN]I[LV][TS][NS][IM]N[MV]TTQxYL | 10 | SlbZIP06,07,10,15,17,18,19,25,26,34,39,48,49,55 |
| V | V | 5 | 31 | 4.0e-104 | ELK[LQ]R[LI]QA[LIM][ESA]Q[QE][AK]QL[RK]D[AV][LE]NEAL[KR][AE]E[LV][EQ]RL[KR] | 3 | SlbZIP45,46,68 |
|  | V | 6 | 16 | 1.6e-122 | QL[EQ][AE]ENxRL[RKS]AQ[LG]xEL | 3 | SlbZIP45,46,68 |
|  | V | 19 | 12 | 1.9e-017 | HRR[SA][HN]S[DE][VST][FI][FA][RF][LF] | 3 | SlbZIP45,46,68 |
|  | V | 27 | 16 | 1.1e-006 | [MQ]A[HQ]LPP[RK][ACV]P[NFT][MQ][TAK][QHK][NP][WL][PT] | 3 | SlbZIP45,46,68 |
| VI | VI | 6 | 16 | 1.6e-122 | QL[EQ][AE]ENxRL[RKS]AQ[LG]xEL | 11 | SlbZIP02,08,09,14,31,33,52,54,56,61,65 |
|  | VI | 7 | 25 | 4.3e-075 | [GQT][QE]R[QE][SAL][TN]LGE[MI]TLE[DE]FLV[KR]AG[VA]V[RA]E[DES] | 11 | SlbZIP02,08,09,14,31,33,52,54,56,61,65 |
|  | VI | 8 | 31 | 9.0e-062 | [GA][GS]SLQ[RS]Q[GS]S[LI]TLPRTLSQKTVDEVWR[DE][IF]QK[EG] | 10 | SlbZIP08,09,14,31,33,52,54,56,61,65 |
|  | VI | 9 | 42 | 1.7e-045 | Q[GAPS]S[IL]YSLT[LF]DE[FVL][QLR][NHST][QTS][LFTV][GCS][GDEN][LSC]GK[DPN][FL][GNS][SN][MI]N[ML][DE]E[LF][LV][KN][STN][IV]W[TN][AIV]E[EAS][NS]Q | 5 | SlbZIP09,33,52,56,61 |
|  | VI | 23 | 19 | 1.0e-013 | [QN][QV]Q[QS][LQS]Q[QH][QH][QP][QP]QQQ[PN][LHI][FMP][PQ][QK]Q | 7 | SlbZIP09,33,52,54,56,61,65 |
|  | VI | 24 | 25 | 2.7e-011 | [NK][LGV][DY][TR][SP][SF][LV]SP[SV]PY[AV][FC][NGS][EG]G[GL]RGR[KR][SY][CGS][ST] | 4 | SlbZIP09,33,54,65 |
| VII | VII | 1 | 50 | 3.7e-336 | V[DE][GN][CV]L[AN]HYD[EN][LI]FR[LI]K[AG][DV]AAK[AS]DVF[HY][LI][LM]SGMW[KR]T[PS][AV]ER[CF]F[LM]W[IL]GGF[RK][PS]SEL | 12 | SlbZIP01,27,28,32,37,44,58,59,60,66,67,69 |
|  | VII | 2 | 50 | 2.1e-313 | F[VL][RN]QAD[NH]LRQQT[LI][QH]Q[ML][HS]RILTTRQ[AS]AR[ACG][LF]LAIG[ED]YFSRLRALSSLW[LA]ARP | 12 | SlbZIP01,27,28,32,37,44,58,59,60,66,67,69 |
|  | VII | 3 | 50 | 1.6e-222 | [LI]K[ILV]L[IM][NP][QH]LEPLT[ED]QQ[LI][VL]G[IV][CY]NL[QR]QS[SC]QQAE[DE]ALSQG[ML]E[KA]LQQSL[AI][ED]T[IL]A[AT] | 12 | SlbZIP01,27,28,32,37,44,58,59,60,66,67,69 |
|  | VII | 4 | 31 | 1.1e-136 | [SN]G[AI][AL]AF[DE]MEY[SA]RW[LV][ED]E[HQ][NQH][RK][LQ][IT][NC][ED]LR[NT]A[LV][NQ]SH | 12 | SlbZIP01,27,28,32,37,44,58,59,60,66,67,69 |
|  | VII | 6 | 16 | 1.6e-122 | QL[EQ][AE]ENxRL[RKS]AQ[LG]xEL | 12 | SlbZIP01,27,28,32,37,44,58,59,60,66,67,69 |
|  | VII | 10 | 21 | 1.7e-032 | [GT][NG][VM][AG]NYM[GS]QM[AV][MIS][AS]M[GEN]KL[GE][TA]LE | 12 | SlbZIP01,27,28,32,37,44,58,59,60,66,67,69 |
| VIII | VIII | 6 | 16 | 1.6e-122 | QL[EQ][AE]ENxRL[RKS]AQ[LG]xEL | 2 | SlbZIP12 |
|  | VIII | 13 | 42 | 4.1e-033 | [NP]F[GH][EHQ][FM]S[SMV][SW][EM]D[AS]F[FR][GN][DE]I[LM][KP][DS][AV][HDG]AC[MT][HIV][AST][HP][TAP][CN][DN][PR][PA][DG][DP][DK][NS][GS][HY]T[HS][TGS][CDE] | 2 | SlbZIP12 |
|  | VIII | 15 | 50 | 2.2e-025 | IEGEIGSFPYQKPMKSGNTYQHIVNPNFPGAYVVNSCNLQCDDQVYCLHP | 2 | SlbZIP12 |
|  | VIII | 21 | 45 | 2.1e-015 | [DG][GF][TV]V[LD]N[GM][QM]G[FT][NG]NC[EQ]F[EP][TV][LA][QM][CQ][LN]G[NP][QG][TG][STY]G[LM]E[EP][VQ]P[GH][CM][VG][VF]GN[SG][TV][PV][TA][DI][NT][TG] | 2 | SlbZIP12 |
| IX | IX | 5 | 31 | 4.0e-104 | ELK[LQ]R[LI]QA[LIM][ESA]Q[QE][AK]QL[RK]D[AV][LE]NEAL[KR][AE]E[LV][EQ]RL[KR] | 7 | SlbZIP11,30,35,40,41,42,43 |
|  | IX | 6 | 16 | 1.6e-122 | QL[EQ][AE]ENxRL[RKS]AQ[LG]xEL | 7 | SlbZIP11,30,35,40,41,42,43 |
|  | IX | 16 | 16 | 8.0e-024 | KK[AI]MAP[DE][KR]LAE[LI][AW][LT]ID | 7 | SlbZIP11,30,35,40,41,42,43 |
|  | IX | 19 | 12 | 1.9e-017 | HRR[SA][HN]S[DE][VST][FI][FA][RF][LF] | 7 | SlbZIP11,30,35,40,41,42,43 |
|  | IX | 23 | 19 | 1.0e-013 | [QN][QV]Q[QS][LQS]Q[QH][QH][QP][QP]QQQ[PN][LHI][FMP][PQ][QK]Q | 7 | SlbZIP11,30,35,40,41,42,43 |
|  | IX | 26 | 11 | 9.4e-010 | RHR[HR]SNS[MV]D[SG]S | 7 | SlbZIP11,30,35,40,41,42,43 |
| X | X | 5 | 31 | 4.0e-104 | ELK[LQ]R[LI]QA[LIM][ESA]Q[QE][AK]QL[RK]D[AV][LE]NEAL[KR][AE]E[LV][EQ]RL[KR] | 2 | SlbZIP63,64 |
|  | X | 19 | 12 | 1.9e-017 | HRR[SA][HN]S[DE][VST][FI][FA][RF][LF] | 2 | SlbZIP63,64 |
| XI | XI | 6 | 16 | 1.6e-122 | QL[EQ][AE]ENxRL[RKS]AQ[LG]xEL | 2 | SlbZIP53,62 |
