# Supplementary material for: Genome-wide systematic characterization of the bZIP transcriptional factor family in tomato (Solanum lycopersicum L.)
Source: BMC Genomics. 2015 Oct 12;16:771. doi: 10.1186/s12864-015-1990-6 (PMC4603586; doi:10.1186/s12864-015-1990-6)
Supplement: Additional file 5: Table S3. — DNA binding specificity prediction of SlbZIP transcription factors for each group. (DOCX 19 kb) [file 12864_2015_1990_MOESM5_ESM.docx]

**Additional file 5: Table S3.** DNA binding specificity prediction of SlbZIP transcription factors for each group

| Group  (No. of Members) | Characteristic Features | Putative Binding Site | Reported Binding Sites of tomato genes |
| --- | --- | --- | --- |
| I (6) | Conserved residues in positions -18 (N),-15 (S), -14 (A), -11 (S), -10 (R) and has RKQS conserved sequence in the basic region. Have a AEC(/A)E(/D)EL hinge sequence specific to GBFs. | G-box and/or G-box-like sequences | LeGBF4, LeGBF9, LeGBF12 |
| II (1) | NRVSAQQAR sequence in basic region | TGACGT-containing and G-box-like sequences | / |
| III (4) | Key residues in the basic region RNR(/K)xS(/A)AxxSR | G- and C-boxes | / |
| IV (20) | Specific hinge region sequence Q(/T/)xH(/Y/L)L(/I)xE(/D) | Hybrid ACGT elements like G/C,G/A,C/G boxes | LebZIP1, LebZIP2, ABZ1 |
| V (3) | Basic region has A (Alanine) residue at -19 position and hinge region has a conserved QYI(/V)SE sequence | unknown | / |
| VI (11) | Conserved motifs M(/K)IK in the basic region and QAY in the hinge region | ABREs with the core ACGT or others containing GCGT or AAGT | SlAREB1, SlAREB2 |
| VII (12) | Conserved residues in positions -21 (L), -20 (A), -19 (Q),-18 (N),-15 (A),-14 (A), -12 (K),-11 (S), and -10 (R). Possess a KAYV(/I)QQ hinge sequence specific to CBFs | GCC binding C-box sequence | / |
| VIII (1) | Conserved residue in position -15 (A) specific to CBFs | C-box elements preferentially | / |
| IX (7) | Conserved K (Lysine) replacement at -10 position of the basic region instead of R (Arginine) | non-palindromic binding site | VSF-1 |
| X (2) | K (Lysine) instead of N (Asparagine) at position -18 | unknown | / |
| XI (2) | Hydrophobic I (Isoleucine) residue at position -10 instead of R (Arginine) or K (Lysine) | unknown | / |
