# Supplementary figures and images for: Genome-wide systematic characterization of the bZIP transcriptional factor family in tomato (Solanum lycopersicum L.)
Source: BMC Genomics. 2015 Oct 12;16:771. doi: 10.1186/s12864-015-1990-6 (PMC4603586; doi:10.1186/s12864-015-1990-6)

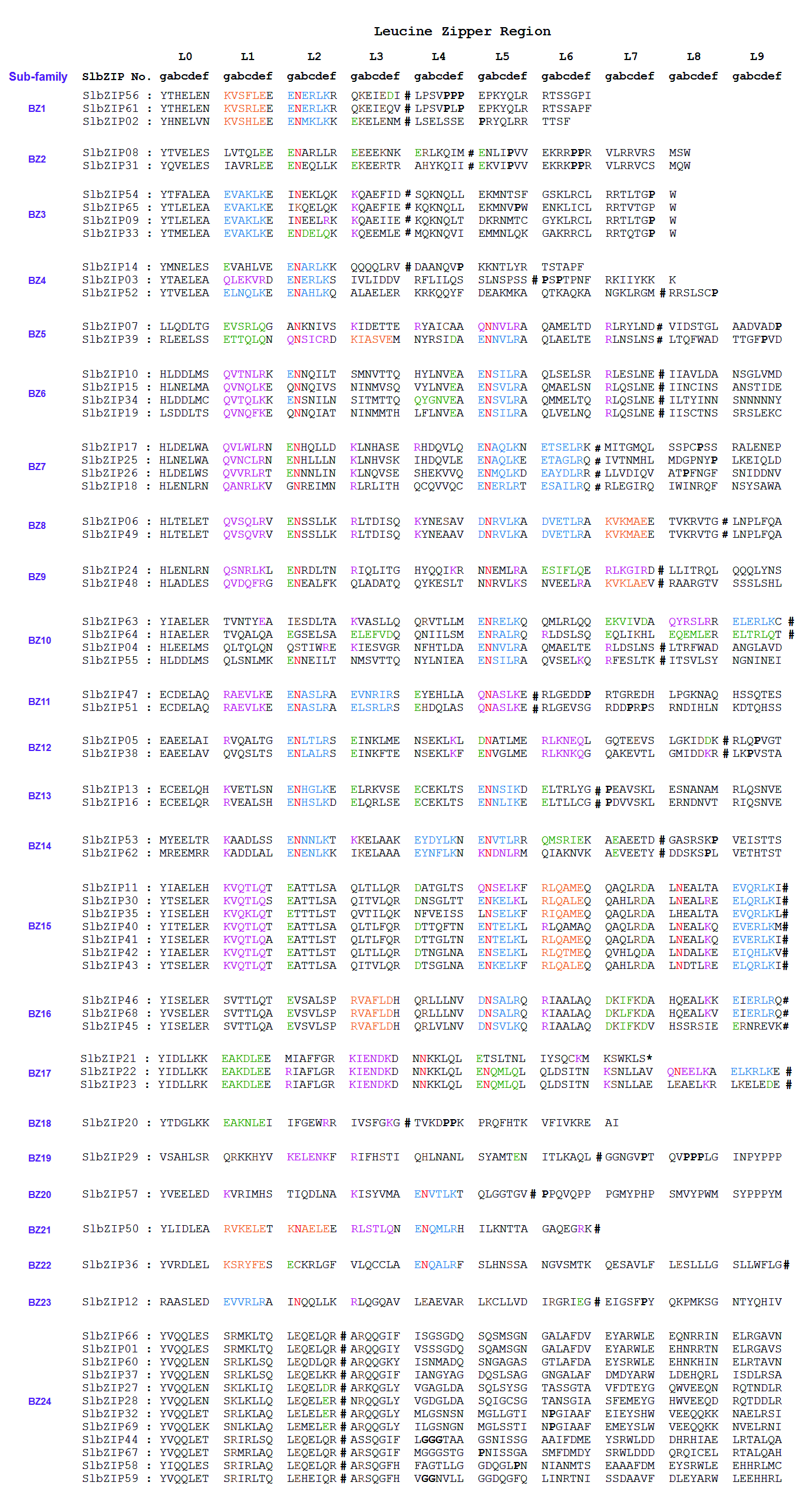

Supplement: Additional file 6: Figure S3. — Amino acid sequence alignment of the leucine zipper region of 69 SlbZIP proteins. The boundaries of the Leu zippers in SlbZIPs were defined according to the criteria used for the Arabidopsis, rice and maize bZIP proteins and the Leu zipper regions were then arranged in the form of heptad repeats, in which the amino acid positions of each heptad was named g, a, b, c, d, e and f in order. Four colors are used to differentiate between different g↔e’pairs. Attractive basic-acidic pairs (R↔E and K↔E) are colored orange, attractive acidic-basic pairs (E↔R, E↔K, D↔R, and D↔K) are blue, repulsive basic pairs (K↔K, R↔K, R↔Q, Q↔K, and K↔Q) are purple and repulsive acidic pairs (E↔E, E↔D, E↔Q, and Q↔E) are green. If only one of the two amino acids in the g↔e’ pair is charged, the residue is colored purple for basic and green for acidic. If the a or d position is charged, it is colored brown. Asparagines at a position are colored red. The prolines and glycines are bold to indicate a potential break in the α-helix. The predicted C-terminal boundary is denoted by the symbol #. (TIFF 14383 kb) [file 12864_2015_1990_MOESM6_ESM.tif]

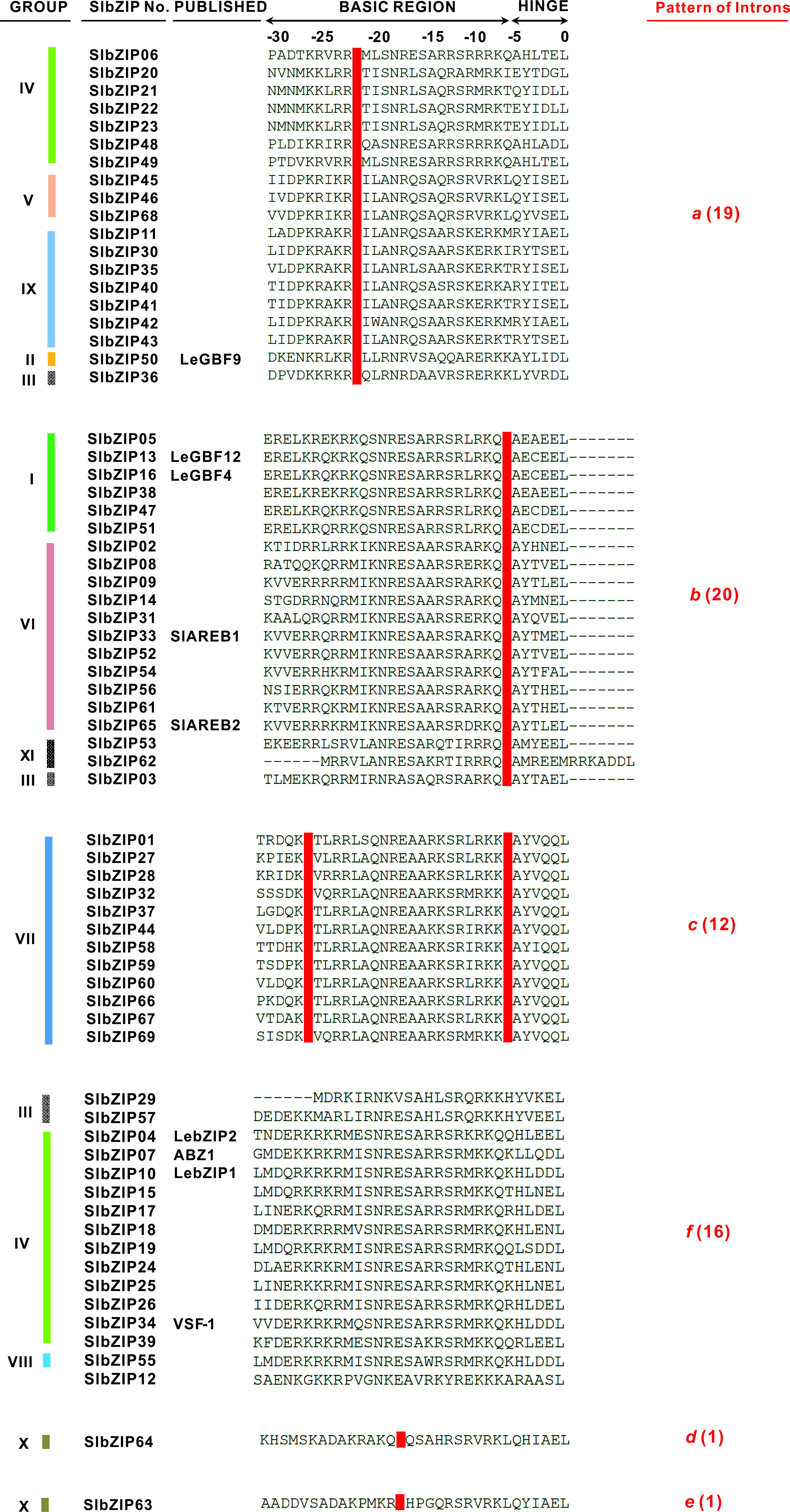

Supplement: Additional file 9: Figure S5. — Position and pattern of introns within the basic and hinge regions of the bZIP domains of the SlbZIP transcription factors. (JPEG 11880 kb) [file 12864_2015_1990_MOESM9_ESM.jpg]
