# Supplementary material for: Genome-wide systematic characterization of the bZIP transcriptional factor family in tomato (Solanum lycopersicum L.)
Source: BMC Genomics. 2015 Oct 12;16:771. doi: 10.1186/s12864-015-1990-6 (PMC4603586; doi:10.1186/s12864-015-1990-6)
Supplement: Additional file 7: Table S4. — Classification of SlbZIP proteins into sub-families with similar predicted dimerization specificity. (DOCX 23 kb) [file 12864_2015_1990_MOESM7_ESM.docx]

**Additional files 7: Table S4.** Classification of SlbZIP proteins into sub-families with similar predicted dimerization specificity.

| **Sub-**  **family** | **No. of Members** | **Heptad with N at** ***a* position** | **Length in heptades** | **Comments** |
| --- | --- | --- | --- | --- |
| BZ1 | 3 | 2 | 3 | Presence of N in *a* position of 2nd heptad and attractive *g*↔*e*′ interactions in the 1st and 2nd heptads as well as lack of any repulsive interactions will favor homo-dimerization between the same SlbZIP protein or within the sub-family. |
| BZ2 | 2 | 2 | 4 | N in a position of 2nd heptad and presence of incomplete *g*↔*e*′ pairs in 1-4 heptad, presence of charged residues in *a* position of 3rd and 4th and *d* position of 3rd heptads. |
| BZ3 | 4 | 2 | 3 | Presence of a single attractive *g*↔*e*′ interaction in the 1st heptad followed by N in *a* position of 2nd heptad (except SlbZIP67) would stabilize homo-dimers, but the probability of hetero-dimerization cannot be ruled out due to the incomplete *g*↔*e*′ pairs in 2nd and/or 3rd heptads. |
| BZ4 | 3 | 2 | 3, 5, 7 | Homo-dimerization within the group favored by an attractive *g*↔*e*′ pair and N in *a* position of 2nd heptad. Incomplete or repulsive *g*↔*e*′ pairs in other heptads suggest the probability of hetero-dimerization |
| BZ5 | 2 | 2, 5 | 7 | Dimerization within the sub-family can be promoted by Ns in *a* position of 2nd and 5th heptads and repulsive *g*↔*e*′ pairs in the 1st, 2nd and 5th heptads. This is supported by an earlier experimental evidence where ABZ1 (SlbZIP07) has been shown to dimerize with itself. Repulsive and incomplete *g*↔*e*′ interactions may also support hetero-dimerization with other SlbZIP proteins. |
| BZ6 | 4 | 2, 5 | 7 | Factors contributing to homo-dimerization are Ns in 2nd and 5th heptad *a* positions and an attractive *g*↔*e*′ interaction in the 5th heptad. Occurrence of repulsive and incomplete *g*↔*e*′ pairs should promote hetero-dimerization. |
| BZ7 | 4 | 2, 5 | 6 | Intra group dimerization should be present due to Ns in 2nd and 5th heptad *a* position and presence of 2 attractive *g*↔*e*′ pairs in 5th and 6th heptads. An incomplete *g*↔*e*′ pair is present which can support intra group as well as inter group dimerization with sub-family BZ8, which shares some of the features present in this sub-family. |
| BZ8 | 2 | 2, 5 | 8 | Sub-family members are expected to dimerize due to Ns in 2nd and 5th heptad *a* position and presence of 2-3 attractive *g*↔*e*′ pairs. Incomplete *g*↔*e*′ pairs are present which can support both homo- and hetero-dimerization. Repulsive *g*↔*e*′ interactions may drive hetero-dimerization with group members like BZ7. |
| BZ9 | 2 | 2, 5 | 7 | Presence of Ns in position of 2nd and 5th heptad. Incomplete or attractive *g*↔*e*′ interactions in most of the heptads should give the potential of promiscuous dimerization to SlbZIP49. But apart from having partial *g*↔*e*′ pairs promoting hetero-dimerization, SlbZIP25, is also expected to homo-dimerize since it has repulsive *g*↔*e*′ pairs in 6th and 7th heptads. |
| BZ10 | 4 | 2, 5 | 7, 9 | Presence of N in a position of 5th heptad and presence of a attractive *g*↔*e*′ pairs in 5th heptads. Interestingly, one member (SlbZIP55) have N in a position of 2nd heptad. Incomplete and other attractive/repulsive *g*↔*e*′ pairs are present which can support both homo- and hetero-dimerization. |
| BZ11 | 2 | 2, 5 | 5 | Expected to homo-dimerize as depicted by the presence of Ns in *a* position of 2nd and 5th heptads and two attractive *g*↔*e*′ pairs in the 2nd and 3rd heptads, one repulsive *g*↔*e*′ pairs in the 5th heptads. Similar *a* position N pattern and incomplete *g*↔*e*′ interactions may lead to hetero-dimer formation with sub-families BZ12, BZ13 and BZ14. |
| BZ12 | 2 | 2, 5 | 8 | Expected to homo-dimerize as depicted by the presence of Ns in *a* position of 2nd and 5th heptads and a attractive and repulsive *g*↔*e*′ pairs in the 2nd and 6th heptads, respectively. Similar *a* position N pattern and incomplete *g*↔*e*′ interactions may lead to hetero-dimer formation with sub-families BZ11, BZ13 and BZ14. |
| BZ13 | 2 | 2, 5 | 6 | Attractive *g*↔*e*′ interactions in the 2nd and 5th heptads and presence of Ns in *a* position of 2nd and 5th heptad point towards homo-dimerization. |
| BZ14 | 2 | 2, 5 | 7 | Attractive *g*↔*e*′ interactions in the 2nd and 4th heptads and presence of Ns in *a* position of 2nd and 5th heptad point towards homo-dimerization. Incomplete and other repulsive *g*↔*e*′ pairs are present which can support both homo- and hetero-dimerization. |
| BZ15 | 7 | 5, 8 | 9 | Presence of Ns in *a* position of 5th and 8th heptads except presence of H not N for SlbZIP35 in heptad 8, along with the presence of attractive *g*↔*e′* interactions in the 5th, 6th and 9th heptads will promote homo-dimerization within the sub-family. Presence of a repulsive *g*↔*e*′ pair in the 1st heptad, incomplete *g*↔*e′* interactions, followed by R or Q in 7th heptad *d* positions are capable of driving hetero-dimerization. This sub-family includes VSF-1 (SlbZIP36) which have been shown to dimerize with each other. |
| BZ16 | 3 | 5 | 9 | Homo-dimers could be formed because of attractive *g*↔*e*′ pairs in 3rd and 9th heptads alongwith N in a position of 5th heptad. Hetero-dimers could also be stabilized due to a repulsive *g*↔*e*′ pair in the 7th heptad, 4 incomplete *g*↔*e*′ pairs and R in 4th heptad *a* position. |
| BZ17 | 3 | 4, 5 | 7, 9 | Ns in position of 4th and 5th heptad (except SlbZIP22) and presence of two repulsive *g*↔*e*′ pair in 1st and 3rd heptad. Other repulsive and attractive *g*↔*e*′ pairs are present which can support both homo- and hetero-dimerization. |
| BZ18 | 1 | / | 3 | Absence of charged residues in *a* position. Incomplete and repulsive *g*↔*e*′ pairs are present in 1st heptad which can support both homo- and hetero-dimerization. |
| BZ19 | 1 | / | 6 | Absence of charged residues in *a* position. Incomplete and repulsive *g*↔*e*′ pairs are present in 2nd heptad which can support both homo- and hetero-dimerization. |
| BZ20 | 1 | 4 | 5 | Presence of a single attractive *g*↔*e*′ interaction and a N in *a* position of 4th heptad would stabilize homo-dimers. |
| BZ21 | 1 | 2, 4 | 6 | N in *a* position of 4th heptad and three attractive *g*↔*e*′ pair in 1st, 2nd and 4th heptad, one repulsive *g*↔*e*′ pair in 3rd heptad may drive homo- and hetero- dimerization. |
| BZ22 | 1 | 4 | 9 | N in *a* position of 4th heptad and two attractive *g*↔*e*′ pair in 1st and 4th heptad. |
| BZ23 | 1 | 2 | 6 | Predominance of hetero-dimer formation features like incomplete *g*↔*e*′ pairs, presence of charged residues in *a* position of 4th and 5th heptads. A single attractive *g↔e*′ pair in 1st heptad and N at a position of 2nd heptad may lead to homo-dimer formation. |
| BZ24 | 12 | / | 2 | Absence of attractive *g*↔e′ interactions as well as presence of charged residues in *a* position indicate destabilization of homo-dimers. Resemble group T of *Arabidopsis* bZIP proteins, which are thought to have an unstable leucine zipper probably stabilized by an additional protein at the time of DNA binding. |
