# Supplementary material for: Genome-wide systematic characterization of the bZIP transcriptional factor family in tomato (Solanum lycopersicum L.)
Source: BMC Genomics. 2015 Oct 12;16:771. doi: 10.1186/s12864-015-1990-6 (PMC4603586; doi:10.1186/s12864-015-1990-6)
Supplement: Additional file 10: Table S5. — Gene clusters of SlbZIP transcription factor family. (DOC 50 kb) [file 12864_2015_1990_MOESM10_ESM.doc]

| **Additional file 10: Table S5.** Gene clusters of SlbZIP transcription factor family | | | | | | |
| --- | --- | --- | --- | --- | --- | --- |
| Cluster | Chr | Name | ID | Chromosome position | Group | Cluster type |
| 1 | 1 | *SlbZIP01* | *Solyc01g008730* | 2,765,457.00 | VII | VII&VI |
|  | 1 | *SlbZIP02* | *Solyc01g008980* | 2,999,069.00 | VI |  |
| 2 | 1 | *SlbZIP10* | *Solyc01g109880* | 88,485,104.00 | IV | IV&IX |
|  | 1 | *SlbZIP11* | *Solyc01g110480* | 88,941,044.00 | IX |  |
| 3 | 3 | *SlbZIP20* | *Solyc03g043810* | 11,449,274.00 | IV | duplication |
|  | 3 | *SlbZIP21* | *Solyc03g043820* | 11,460,790.00 | IV |  |
|  | 3 | *SlbZIP22* | *Solyc03g043830* | 11,477,154.00 | IV |  |
|  | 3 | *SlbZIP23* | *Solyc03g043840* | 11,482,707.00 | IV |  |
|  | 3 | *SlbZIP24* | *Solyc03g043900* | 11,579,314.00 | IV |  |
| 4 | 4 | *SlbZIP30* | *Solyc04g071160* | 55,678,517.00 | IX | IX&VI |
|  | 4 | *SlbZIP31* | *Solyc04g071510* | 56,088,746.00 | VI |  |
| 5 | 4 | *SlbZIP34* | *Solyc04g080740* | 62,438,744.00 | IV | IV&IX |
|  | 4 | *SlbZIP35* | *Solyc04g081190* | 62,785,114.00 | IX |  |
| 6 | 9 | *SlbZIP52* | *Solyc09g009490* | 2,914,302.00 | VI | VI&XI |
|  | 9 | *SlbZIP53* | *Solyc09g009760* | 3,246,743.00 | XI |  |
| 7 | 10 | *SlbZIP56* | *Solyc10g076920* | 59,213,423.00 | VI | III&VI&VII |
|  | 10 | *SlbZIP57* | *Solyc10g078290* | 59,447,849.00 | III |  |
|  | 10 | *SlbZIP58* | *Solyc10g078670* | 59,732,997.00 | VII |  |
| 8 | 10 | *SlbZIP59* | *Solyc10g080410* | 61,015,000.00 | VII | VI&VII |
|  | 10 | *SlbZIP60* | *Solyc10g080770* | 61,293,121.00 | VII |  |
|  | 10 | *SlbZIP61* | *Solyc10g081350* | 61,775,875.00 | VI |  |
