# Supplementary material for: Genome-wide systematic characterization of the bZIP transcriptional factor family in tomato (Solanum lycopersicum L.)
Source: BMC Genomics. 2015 Oct 12;16:771. doi: 10.1186/s12864-015-1990-6 (PMC4603586; doi:10.1186/s12864-015-1990-6)
Supplement: Additional file 11: Table S6. — SlbZIP genes present on duplicated chromosomal segments. (DOC 70 kb) [file 12864_2015_1990_MOESM11_ESM.doc]

| **Additional file 11: Table S6***. SlbZIP* genes present on duplicated chromosomal segments. | | | | | | | |
| --- | --- | --- | --- | --- | --- | --- | --- |
| **Chromosome** | ***SlbZIP* No.** | **Coordinates** | **Chromosome** | ***SlbZIP* No.** | **Coordinates** | **BLASTP E-value** | **Group** |
| 1 | SlbZIP01 | 2772962 2765457 | 10 | SlbZIP60 | 61293121 61301987 | 5.00E-119 | VII |
| 1 | SlbZIP01 | 2772962 2765457 | 11 | SlbZIP66 | 47352691 47346074 | 2.00E-134 | VII |
| 1 | SlbZIP02 | 2995222 2999069 | 10 | SlbZIP61 | 61775875 61780773 | 1.00E-25 | VI |
| 1 | SlbZIP04 | 71029930 71029496 | 1 | SlbZIP07 | 82244245 82244661 | 1.00E-25 | IV |
| 1 | SlbZIP04 | 71029930 71029496 | 6 | SlbZIP39 | 3585802 3585368 | 1.00E-57 | IV |
| 1 | SlbZIP05 | 78465956 78469714 | 5 | SlbZIP38 | 59415665 59420281 | 5.00E-135 | I |
| 1 | SlbZIP09 | 87202306 87200159 | 4 | SlbZIP33 | 61097157 61094342 | 2.00E-76 | VI |
| 1 | SlbZIP09 | 87202306 87200159 | 10 | SlbZIP54 | 43596753 43594760 | 8.00E-105 | VI |
| 1 | SlbZIP10 | 88485104 88485595 | 2 | SlbZIP15 | 42509803 42509369 | 5.00E-37 | IV |
| 1 | SlbZIP10 | 88485104 88485595 | 4 | SlbZIP34 | 62438744 62439187 | 1.00E-33 | IV |
| 1 | SlbZIP11 | 88941044 88943305 | 4 | SlbZIP35 | 62785114 62787838 | 7.00E-87 | IX |
| 1 | SlbZIP10 | 89569192 89572825 | 10 | SlbZIP55 | 52389038 52390619 | 7.00E-92 | IV |
| 2 | SlbZIP13 | 29474684 29468336 | 2 | SlbZIP16 | 42509803 42509369 | 4.00E-95 | I |
| 2 | SlbZIP15 | 41465770 41466655 | 2 | SlbZIP13 | 28138501 2813930 | 4.00E-22 | I |
| 2 | SlbZIP15 | 42509803 42509369 | 3 | SlbZIP19 | 9611703 9611356 | 1.00E-34 | IV |
| 2 | SlbZIP15 | 42509803 42509369 | 4 | SlbZIP34 | 62438744 62439187 | 8.00E-36 | IV |
| 2 | SlbZIP17 | 45834988 45835530 | 3 | SlbZIP25 | 16750062 16749270 | 6.00E-48 | IV |
| 2 | SlbZIP17 | 45834988 45835530 | 4 | SlbZIP26 | 118622 118047 | 2.00E-37 | IV |
| 2 | SlbZIP18 | 9611703 9611356 | 3 | SlbZIP24 | 11579314 11578703 | 2.00E-41 | IV |
| 3 | SlbZIP19 | 11449274 11444579 | 4 | SlbZIP34 | 62438744 62439187 | 1.00E-24 | IV |
| 4 | SlbZIP27 | 4150679 4154818 | 4 | SlbZIP28 | 51450108 51444726 | 1.00E-150 | VII |
| 4 | SlbZIP28 | 53387307 53389172 | 4 | SlbZIP32 | 57075743 57078296 | 4.00E-100 | VII |
| 6 | SlbZIP44 | 42332726 42337922 | 11 | SlbZIP67 | 50210714 50219552 | 1.00E-145 | VII |
| 7 | SlbZIP45 | 59220996 59219093 | 7 | SlbZIP46 | 62620405 62618690 | 2.00E-61 | V |
| 9 | SlbZIP53 | 3246743 3241071 | 10 | SlbZIP62 | 62534173 62535719 | 4.00E-21 | XI |
| 9 | SlbZIP45 | 3246743 3241071 | 12 | SlbZIP68 | 3717269 3715930 | 3.00E-76 | V |
| 10 | SlbZIP56 | 59213423 59214274 | 10 | SlbZIP61 | 61775875 61780773 | 4.00E-89 | VI |
| 10 | SlbZIP58 | 59732997 59726600 | 10 | SlbZIP59 | 61015000 61008679 | 2.00E-168 | VII |
