# Supplementary material for: Genome-wide systematic characterization of the bZIP transcriptional factor family in tomato (Solanum lycopersicum L.)
Source: BMC Genomics. 2015 Oct 12;16:771. doi: 10.1186/s12864-015-1990-6 (PMC4603586; doi:10.1186/s12864-015-1990-6)
Supplement: Additional file 12: Table S7. — Primers used in this study. (DOC 132 kb) [file 12864_2015_1990_MOESM12_ESM.doc]

**Additional file 12: Table S7. Primers used in this study**

| Primers | Sequences (5’-3’) | Size (bp) |
| --- | --- | --- |
| *qRT-PCR* | | |
| SlbZIP01-q-1F | ACCAAAAGACGCTTCGTAGGC | 130 |
| SlbZIP01-q-1R | TGAAGCTCCTGCTCTAGCTGTG |
| SlbZIP02-q-1F | TGAGGAGATGAACAGCCAGGAG | 96 |
| SlbZIP02-q-1R | GAAGCATCAGCAACGAAAAGC |
| SlbZIP04-q-1F | GCTTCGACTCAGCAACCAGCTA | 136 |
| SlbZIP04-q-1R | CCAAATGCTGCTGCTTCCTC |
| SlbZIP05-q-1F | AGCGGACCAGAGCAATCTCCAT | 126 |
| SlbZIP05-q-1R | TAAGGGTGAGGGGTATGACCAG |
| SlbZIP06-q-1F | TGATGAATCGCAGCTCTTCCG | 91 |
| SlbZIP06-q-1R | TGAGGAGGAGATGAAGTGGTCG |
| SlbZIP07-q-1F | ATTTCCAACAGGGAATCTGCG | 80 |
| SlbZIP07-q-1R | CTGCTCACTTCCCCTGTCAAA |
| SlbZIP08-q-1F | CAACAACGCCTAATTCGGATCG | 147 |
| SlbZIP08-q-1R | AACAAGCAAAAGGGTCGGAAT |
| SlbZIP09-q-1F | AGCGAAGGCGTAGGAGGATGAT | 138 |
| SlbZIP09-q-1R | CCTGTTTCTTCCGCAACTCTTC |
| SlbZIP10-q-1F | TTGGATGCAAACAGTGGACTTG | 147 |
| SlbZIP10-q-1R | GACATCGGCAGTCATAATGGG |
| SlbZIP11-q-1F | TAAATGGCTGGTGACAATGACG | 139 |
| SlbZIP11-q-1R | TTGTCAACTGGGGTATGTCCAA |
| SlbZIP12-q-1F | TGTAACCCGCCTGGTCCTGATA | 141 |
| SlbZIP12-q-1R | CGGGCGTTTCTTTCCTTTATTC |
| SlbZIP13-q-1F | TACACCTCCCTTCTTTCCCTCA | 95 |
| SlbZIP13-q-1R | GGGCTCCATAAGGAGGCATAA |
| SlbZIP14-q-1F | AAGCAGCAGCAACAGTTACGAG | 89 |
| SlbZIP14-q-1R | TCTCAAAATGGAGCAGTTGACG |
| SlbZIP15-q-1F | GGGAATCAGCAAGAAGGTCAAG | 179 |
| SlbZIP15-q-1R | CAACTCTGCCATTTGTGCTCTG |
| SlbZIP16-q-1F | CCGGCAAATCCTGAAGCAGA | 89 |
| SlbZIP16-q-1R | TGCCTTACCACCAGAACAGACC |
| SlbZIP17-q-1F | GAGCTTTGGGCACAGGTTCTT | 78 |
| SlbZIP17-q-1R | GCGCTCAGAGGCATGATTTAGT |
| SlbZIP18-q-1F | CCGGCATTTGATGGAGGAT | 79 |
| SlbZIP18-q-1R | CAACCGATTCCACTTGTTGTGA |
| SlbZIP24-q-1F | TTTCCGAACACCTTCCCTTCT | 128 |
| SlbZIP24-q-1R | GGTTCAGGAGTTGGGGATTGT |
| SlbZIP26-q-1F | TCGGATGAATCGGAGGAACA | 112 |
| SlbZIP26-q-1R | GGTGCCTTTGTTTCCTCATCC |
| SlbZIP27-q-1F | ATGTGAGCCGATCCATCAAATT | 119 |
| SlbZIP27-q-1R | CCATCATCGGTATCTGGTCCTC |
| SlbZIP28-q-1F | CAGATTGGCATGTGGGGAGA | 77 |
| SlbZIP28-q-1R | TCGAGGCAATTCTCGACTTCA |
| SlbZIP30-q-1F | TCGGATTGACCTTGACCAGATG | 79 |
| SlbZIP30-q-1R | GAAGAAAGTCTCCGACTGTGCC |
| SlbZIP31-q-1F | GATCGAAGGAAATCGTCGGC | 131 |
| SlbZIP31-q-1R | TCAACCGTCATGGAAGTCGC |
| SlbZIP32-q-1F | CCCACAAGAAGGATGGGCATA | 132 |
| SlbZIP32-q-1R | CCCGACTTGTCATCTGGCCTAT |
| SlbZIP33-q-1F | TTAGCTCAACAGGAGGGAGTGG | 132 |
| SlbZIP33-q-1R | GAACACCAACCTCTTGCCCAT |
| SlbZIP34-q-1F | CTGGGTTCTTTGTCTTTGTGGC | 94 |
| SlbZIP34-q-1R | GCCAAAACCCAGAAATCAGAGG |
| SlbZIP35-q-1F | GATGGTGTCGGGTCATTCTAGC | 99 |
| SlbZIP35-q-1R | CCATCCCTCGTAAACGAAGACA |
| SlbZIP36-q-1F | ATTCCTCCCCACAGTCATTCC | 91 |
| SlbZIP36-q-1R | TCGGCAGCAAACTGAGGATC |
| SlbZIP37-q-1F | GCACGCTTTGCTGATATTGGA | 113 |
| SlbZIP37-q-1R | CCAACATTGTTTGGCCTCATTT |
| SlbZIP38-q-1F | ATGCCCATCGAGTAGCTGTACC | 105 |
| SlbZIP38-q-1R | AAGGTGGAAACATAGGCGGTAG |
| SlbZIP39-q-1F | ATGTCTTCGACTCCGCACTTG | 130 |
| SlbZIP39-q-1R | GCTTCTTCATCCGTGACCTCTT |
| SlbZIP40-q-1F | ATCCGTCCAATCCAAACTCCA | 149 |
| SlbZIP40-q-1R | AGACGCATCGAATGGGTCAG |
| SlbZIP41-q-1F | GCAAGATCCGTCAAATCCAAA | 104 |
| SlbZIP41-q-1R | GGAAATTGACCTCCGAGTGAGC |
| SlbZIP42-q-1F | CTCCCTTAGGACCTGGAAATGC | 76 |
| SlbZIP42-q-1R | CTAAACAGGCTTGAATCTGCCG |
| SlbZIP43-q-1F | AAAGCCCATTCAGGCCCAATT | 128 |
| SlbZIP43-q-1R | CATCAAAACTGGGAAAACGGA |
| SlbZIP45-q-1F | TCATCATCAGAACAACCCGTCA | 76 |
| SlbZIP45-q-1R | TCTTCGATGAGACCCTCGTTTT |
| SlbZIP46-q-1F | GCCTTTTCAAAACATGCCTCC | 88 |
| SlbZIP46-q-1R | GAGGAAATCGTCTACCCAGGAA |
| SlbZIP47-q-1F | TCAAAAGAGGCGAAGGAAGCA | 107 |
| SlbZIP47-q-1R | GCCTGAAAGCCAGACCAATCT |
| SlbZIP48-q-1F | GAAATTGGCTGAGGTTAGGGC | 178 |
| SlbZIP48-q-1R | AATTCCACCCCACGAGTCTTC |
| SlbZIP49-q-1F | TGCTGTCCCTGAACATGATGAC | 85 |
| SlbZIP49-q-1R | CGCGTTTTGCATTCTACAATCA |
| SlbZIP50-q-1F | TGCCGCTAGTTCACTACCTTCA | 149 |
| SlbZIP50-q-1R | TTCCAGAAGCTGACGTTGTCCC |
| SlbZIP51-q-1F | ACGAAGACTCCCAATTCACAGG | 123 |
| SlbZIP51-q-1R | AGGACTTGAAGCCATGAACCC |
| SlbZIP53-q-1F | AAGGCGGAGGAAGGGAAGTT | 82 |
| SlbZIP53-q-1R | CCAATGCTTGTGCTGCTTCC |
| SlbZIP54-q-1F | GTTTCCGAAAGGATGTTGGTTC | 127 |
| SlbZIP54-q-1R | AAAGTCCCCGCAGCCATACT |
| SlbZIP55-q-1F | AGGATCGGGGTCAGGGTCATAT | 130 |
| SlbZIP55-q-1R | CCTCATTCTCGATCTCCAAGCT |
| SlbZIP56-q-1F | AATTGGGGAATTGTGGGAAAC | 122 |
| SlbZIP56-q-1R | TTGCTGCACTGGCCCGTAAT |
| SlbZIP57-q-1F | TGGTAATCAGGCAGCAGGAGGT | 104 |
| SlbZIP57-q-1R | ACGACGGCGACACTTCATTC |
| SlbZIP58-q-1F | GCCATCATCCACAAAGGGAA | 187 |
| SlbZIP58-q-1R | TGCATTTTGCTCTGAACCTGAA |
| SlbZIP59-q-1F | CCTCGTCAACAACAACAAATGG | 80 |
| SlbZIP59-q-1R | AAGTTCCCAGGATTAGCTGACG |
| SlbZIP60-q-1F | ATGGGCACAGAAGGGAACAA | 71 |
| SlbZIP60-q-1R | CCAAGATATTGCTCAAGCACCC |
| SlbZIP61-q-1F | TGCTTTGGCTAGGCAAGGAT | 168 |
| SlbZIP61-q-1R | AACACCCCGTAATCCGTTCC |
| SlbZIP62-q-1F | CGGACAATTCGTCGAAGACAG | 110 |
| SlbZIP62-q-1R | TCCGCAGCAGCAAGTTCTTTA |
| SlbZIP63-q-1F | GCCTCGTTGCCCATTTCAGA | 99 |
| SlbZIP63-q-1R | TGATGTCGAGGAGGCAACAAG |
| SlbZIP64-q-1F | GGGGCTACCTCCCTCTTTGATT | 99 |
| SlbZIP64-q-1R | CCATGATCGACTGCTTGTTCG |
| SlbZIP65-q-1F | AAACTTGTACCTGCCAAAACGG | 165 |
| SlbZIP65-q-1R | TGCAGCTCATCAAAGGTAAACG |
| SlbZIP66-q-1F | TCTGATGGAAGTGACAGACCGA | 71 |
| SlbZIP66-q-1R | CGAGCAGCTTCACGATTTTGAG |
| SlbZIP67-q-1F | CGAAAGTTTCGTCCATCAGGC | 154 |
| SlbZIP67-q-1R | ACGTGATGCCCATAGGGAACT |
| SlbZIP68-q-1F | CACCAAGAGCGCCTAACATGA | 154 |
| SlbZIP68-q-1R | GAGTCGCTCATTGATCTTCGGT |
| SlbZIP69-q-1F | TATGAGTATGTGGGAAGACGCG | 91 |
| SlbZIP69-q-1R | GTCATCTGCCCTGTCATTTGC |
| *Transcription activation assay in yeast* | | |
| SlbZIP06-pBD-F | GGGCCTCGAGCCCGGGTCGACATGGATAGGGTATTTTCAGTGGACG | 1365 |
| SlbZIP06-pBD-R | CCGGAATTAGCTTGGCTGCAGTTATTGCTCTCCCCGACCTTGTTATTGCTCTCCCCGACCTTG |
| SlbZIP12-pBD-F | CCC GAATTC ATGGCTGACGGGGAGCTG | 813 |
| SlbZIP12-pBD-R | GGG GTCGAC TCAACTTGTTGTACGCGCGC |
| SlbZIP16-pBD-F | CCC GTCGAC ATGCCTCCTTATGGGACTCC | 855 |
| SlbZIP16-pBD-R | GGG CTGCAG TTAACTAGCTTCTTCAACATTAGAT |
| SlbZIP32-pBD-F | CCC GTCGAC ATGACTTCTCCAACAACTCAAT | 1086 |
| SlbZIP32-pBD-R | GGG CTGCAG TTAGGTAGGTTCACGAGGAC |
| SlbZIP46-pBD-F | CCC GTCGAC ATGGCTCAACTACCACCTAAAG | 909 |
| SlbZIP46-pBD-R | GGG CTGCAG TTACGCTTCTTCCGTACAACTC |
| *Subcellular Localization* | | |
| SlbZIP06-GFP-F | CCCGGATCC ATGGATAGGGTATTTTCAGTGG | 1365 |
| SlbZIP06-GFP-R | CCCTCTAGA TTATTGCTCTCCCCGACCTTG |
| SlbZIP12-GFP-F | CCCGGATCC ATGGCTGACGGGGAGCTG | 813 |
| SlbZIP12-GFP-R | CCCTCTAGA TCAACTTGTTGTACGCGCGC |
| SlbZIP16-GFP-F | CCCGGATCC ATGCCTCCTTATGGGACTCC | 855 |
| SlbZIP16-GFP-R | CCCTCTAGA TTAACTAGCTTCTTCAACATTAGAT |
| SlbZIP32-GFP-F | CCCGGATCC ATGACTTCTCCAACAACTCAAT | 1086 |
| SlbZIP32-GFP-R | CCCTCTAGA TTAGGTAGGTTCACGAGGAC |
| SlbZIP46-GFP-F | CCCGGATCC ATGGCTCAACTACCACCTAAAG | 909 |
| SlbZIP46-GFP-R | CCCTCTAGA TTACGCTTCTTCCGTACAACTC |
